# Exploration of the tunability of BRD4 degradation by DCAF16 *trans*-labelling covalent glues

**DOI:** 10.1016/j.ejmech.2024.116904

**Published:** 2024-09-24

**Authors:** Muhammad Murtaza Hassan, Yen-Der Li, Michelle W. Ma, Mingxing Teng, Woong Sub Byun, Kedar Puvar, Ryan Lumpkin, Brittany Sandoval, Justine C. Rutter, Cyrus Y. Jin, Michelle Y. Wang, Shawn Xu, Anna M. Schmoker, Hakyung Cheong, Brian J. Groendyke, Jun Qi, Eric S. Fischer, Benjamin L. Ebert, Nathanael S. Gray

**Affiliations:** a Department of Chemical and Systems Biology, ChEM-H and Stanford Cancer Institute, Stanford School of Medicine, Stanford University, Stanford, CA, USA; b SPARK Translational Research Program, Stanford University School of Medicine, Stanford, CA, USA; c Department of Molecular and Cellular Biology, Harvard University, Cambridge, MA, USA; d Department of Medical Oncology, Dana-Farber Cancer Institute, Boston, MA, USA; e Cancer Program, Broad Institute of MIT and Harvard, Cambridge, MA, USA; f Department of Cancer Biology, Dana-Farber Cancer Institute, Boston, MA, USA; g Department of Biological Chemistry and Molecular Pharmacology, Harvard Medical School, Boston, MA, USA; h Center for Drug Discovery, Department of Pathology & Immunology, and Verna and Marrs McLean Department of Biochemistry and Molecular Pharmacology, Baylor College of Medicine, Houston, TX, USA; i Howard Hughes Medical Institute, Boston, MA, USA

## Abstract

Chemically induced proximity modalities such as targeted protein degradation (TPD) hold promise for expanding the number of proteins that can be manipulated pharmacologically. However, current TPD strategies are often limited to proteins with preexisting ligands. Molecular glues (*e.g*. glutarimide ligands for CUL4^CRBN^), offer the potential to target undruggable proteins. Yet, their rational design is largely unattainable due to the unpredictability of the ‘gain-of-function’ nature of the glue interaction upon chemical modification of ligands. We recently reported a covalent *trans*-labelling glue mechanism which we named ‘Template-assisted covalent modification’, where an electrophile decorated BRD4 inhibitor was effectively delivered to a cysteine residue on DCAF16 due to an electrophile-induced BRD4-DCAF16 interaction. Herein, we report our efforts to evaluate how various electrophilic modifications to the BRD4 binder, JQ1, affect DCAF16 recruitment and subsequent BRD4 degradation efficiency. We discovered a moderate correlation between the electrophile-induced BRD4-DCAF16 ternary complex formation and BRD4 degradation. Moreover, we show that a more solvent-exposed warhead presentation optimally recruits DCAF16 and promotes BRD4 degradation. The diversity of covalent attachments in this class of BRD4 degraders suggests a high tolerance and tunability for the BRD4-DCAF16 interaction. This offers a new avenue for rational glue design by introducing covalent warheads to known binders.

## Graphical Abstract

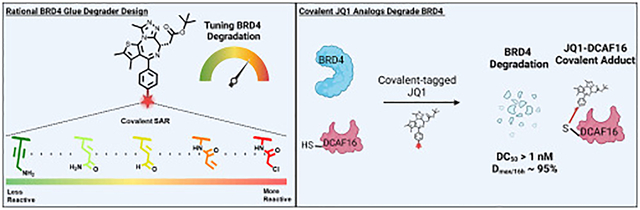

## 1. Introduction

Chemically induced protein degradation has attracted significant interest owing to its unique pharmacology and modular design strategy [1–5]. Unlike the traditional ‘occupancy-driven’ pharmacology, targeted protein degradation (TPD) offers a catalytic ‘event-driven’ pharmacology. Furthermore, degraders have shown to have a potential target scope beyond protein targets that are typically perceived as druggable [5,6]. Traditional inhibitors require deep lipophilic protein pockets suitable for ligand-binding, which hinders inhibitor development for protein targets containing smooth, shallow, and featureless surfaces lacking ligand-protein complementarity [7–9]. Degraders, however, are capable of harnessing protein-protein surface complementarity, which expands the target scope to proteins previously considered undruggable, such as transcription factors [10–13].

TPD strategies which exploit the ubiquitin-dependent proteolysis pathway are commonly classified into two main chemical degrader categories: Proteolysis Targeting Chimeras (PROTACs) and molecular glue degraders (MGDs) [14,15]. PROTACs are heterobifunctional molecules that contain an E3 ligase binder tethered via a linker to a protein-of-interest (POI) binder. Although PROTACs offer a modular design strategy, their target scope is generally limited to POIs that are ligandable and thereby precludes access to many targets considered ‘undruggable’. Recent advances in non-small molecule TAC approaches such as TRAFTACs (Transcription Factor Targeting Chimeras), RNA-PROTAC, and BioPROTAC have expanded the degradable proteome to undruggable targets such as transcription factors, RNA-binding proteins, and other featureless proteins (UBE2C, RAS, etc.) [16]. However, unlike traditional PROTACs and other TAC technologies, molecular glues are monovalent small molecules that can alter a protein’s interactome to induce protein-protein interactions with new protein-partners, thereby expanding their target scope to undruggable proteins. Since MGD chemical design is not plug-and-play by nature, their discovery has largely been serendipitous.

**Fig. 1.**
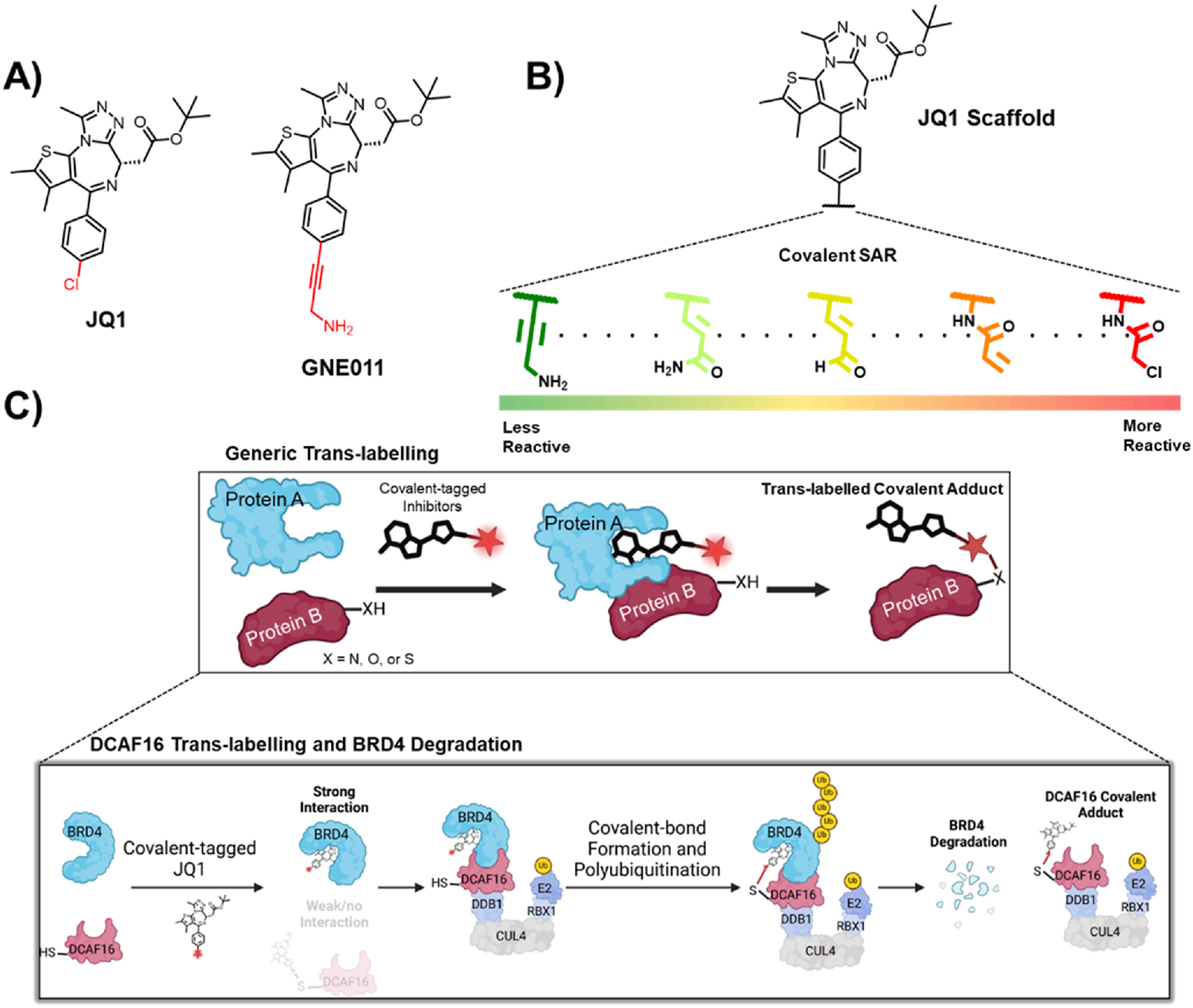
A) The chemical structures of the BRD4 inhibitor JQ1 and its related analog GNE011. B) Investigating various cysteine reactive electrophilic handles. C) A schematic depiction of a general covalent *trans*-labelling event and its relevance to our previous study showing the degradation of BRD4 by covalently *trans-*labelling DCAF16.

Recently we reported MGD that use minimalistic covalent handles. Previous studies had reported the degradation of BRD4 by GNE011, an analog of the BRD4 selective inhibitor JQ1 (Fig. 1A) [17]. Studies by our group and others showed that GNE011 induces the degradation of BRD4 via recruitment of the DDB1 and CUL4 associated factor 16 (DCAF16) E3 ligase to bromodomain 2 (BRD4_BD2_) [18–20]. Via a series of electrophilic substitutions of the propargyl amine tail of GNE011, we discovered that covalent warhead attachment to JQ1 can lead to improved BRD4 degradation compared to GNE011. Further mechanistic and structural analyses uncovered a novel mechanism of action which we termed ‘template-assisted covalent modification’ where BRD4 degradation required the covalent *trans*-labelling of Cys58 on DCAF16. A cryo-electron microscopy (cryo-EM) structure of BRD4_BD2_-MMH2-DCAF16-DDB1 showed structural complementarity between BRD4_BD2_ and DCAF16, and suggested that upon binding to covalent JQ1 analogs, BRD_BD2_ serves as a structural template to facili-tate DCAF16 covalent modification (Fig. 1C) [19].

Herein, we describe the medicinal chemistry campaign that led to the discovery of the covalent JQ1 analogs and explore the relationship between BRD4 degradation with covalent-warhead substitution (Fig. 1B).

## 2. Results

### 2.1. Covalent analogs show tunability of DCAF16-dependent BRD4_BD2_ degradation

**Fig. 2.**
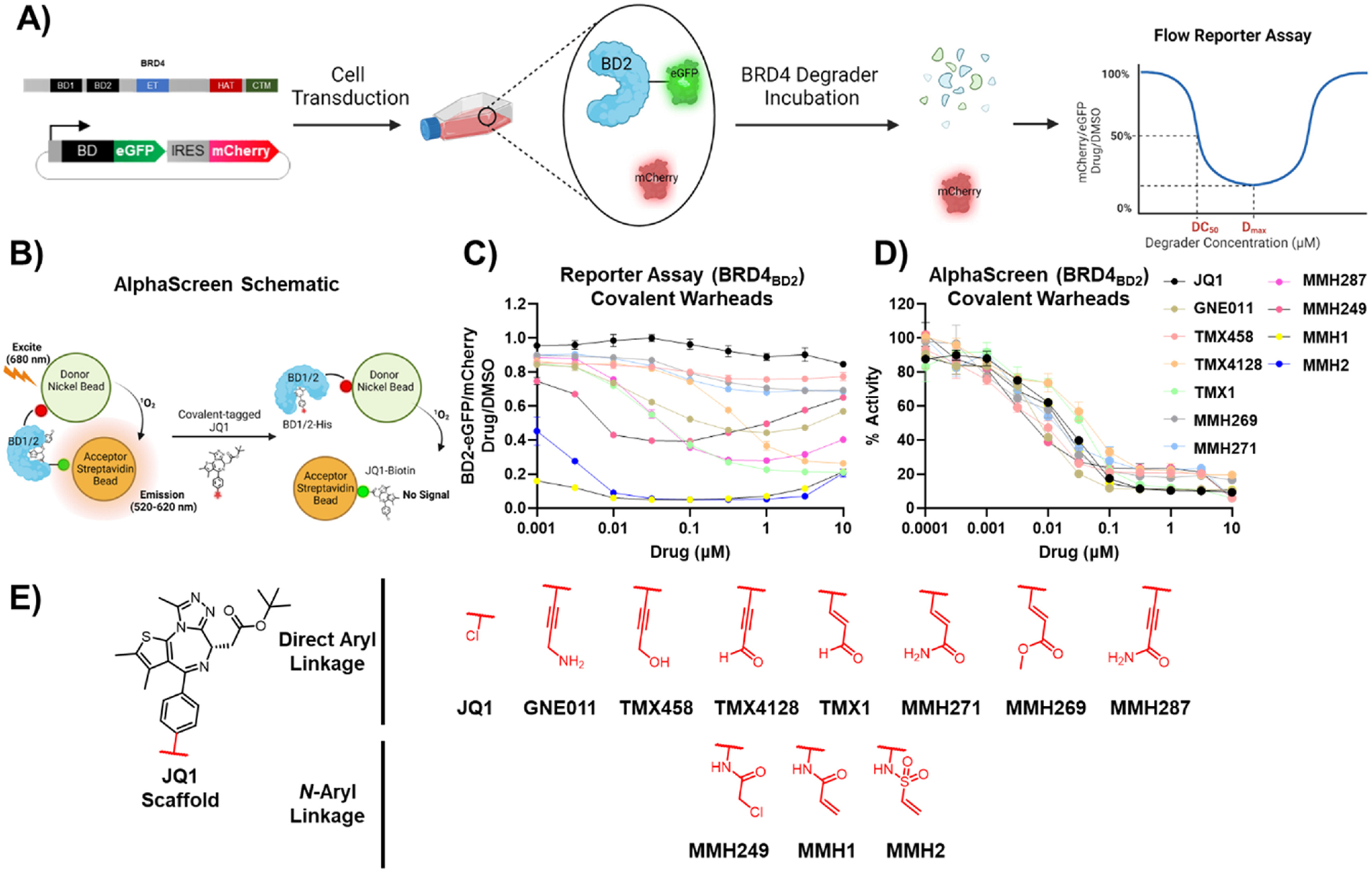
A) A schematic depiction of the BRD4_BD2_-eGFP and mCherry reporter assay and B) an AlphaScreen assay in the context of BRD4 degraders. C) Dose-dependent BRD4_BD2_ degradation by covalent JQ1 analogs as determined by the BRD4_BD2_-eGFP and mCherry reporter assay. D) The dose-dependent inhibition of BRD4_BD2_ by covalent JQ1 analogs as determined by an AlphaScreen assay. E) The chemical structures of the covalent JQ1 degradation tails included in the covalent SAR. Non-covalent analogs are not included. Note: MMH287, and MMH2 were not tested for AlphaScreen BRD4_BD2_.

In order to investigate the degradation of BRD4 by GNE011 and related analogs, we used a fluorescent stability reporter assay reported previously (Fig. 2A) [17]. This assay involves the co-expression of mCherry with BRD4_BD1/2_ fused to an enhanced green fluorescent protein (eGFP) tag, and the subsequent fluorescence measurement of BRD4_BD1/2_-eGFP normalized to mCherry as a readout for BRD4_BD1/2_ degradation. It allows for a quantitative, rapid, and facile screening of several MGDs, in a dose-and time-dependent manner. Hence, this assay was first used to test GNE011 with a 16 h incubation in K562 cells. In this assay, GNE011 showed selective degradation of the second bromodomain (BRD4_BD2_) over the first (BRD4_BD1_) with ~50 % maximal degradation (D_max/16h_) at 16 h (Fig. 2C) [19]. GNE011 was confirmed by us and others, to be degraded via recognition by DCAF16 [18–20]. Thus, the BRD4_BD2_ stability system was used to test all subsequent GNE011 analogs in wild type and *DCAF16*−*/*− cells.

Next, to understand the role of the propargyl amine group for GNE011 induced BRD4 degradation, we introduced propargyl alcohol (TMX458) and propargyl aldehyde (TMX4128) to JQ1. TMX458 and TMX4128 showed disparate reactivities, with TMX458 being completely inactive (D_max/16h_ = 10 %, DC_50_ > 10 μM) and TMX4128 showing enhanced BRD4 degradation in comparison to GNE011 (D_max/16h_ = 69 %, DC_50_ = 703 nM). Given the literature precedence of propargyl aldehydes and alkynes as covalent warhead targeting thiols, we suspected the degradation might be linked to the covalency of GNE011 and its analogs [21–23]. Furthermore, drugs with propargyl amine handles such as rasagiline and selegiline are reported to irreversibly inhibit monoamine oxidase enzymes [24–27]. Hence, we focused our subsequent structural activity relationship (SAR) studies to survey various covalent warhead attachments (Fig. 1b).

First, we designed a series of Michael acceptor chemotypes, including acrolein (TMX1), methyl acrylate (MMH269), aryl acrylamide (MMH271), and propiolamide (MMH287). MMH269 and MMH271 showed almost no DCAF16 dependent BRD4_BD2_ degradation, whereas TMX1 and MMH287 were essentially equipotent with respect to their D_max/16h_ (75 % and 69 %) and DC_50_ values (64 nM and 51 nM; Fig. S1).

Next, we wanted to confirm whether covalent bond formation was a requirement for DCAF16 dependent BRD4_BD2_ degradation. Intact mass spectrometry and mutagenesis experiments validated the mass adduct formation of TMX1-DCAF16 upon co-incubation with BRD4_BD2_ and identified DCAF16 Cys58 as the targeted residue. Moreover, mutagenesis studies using a CRISPR alanine screen (C58A), and TR-FRET experiments (C58S) showed a complete rescue of BRD4 degradation and DCAF16 recruitment respectively confirming the necessity for DCAF16 covalent-bond formation for BRD4 degradation [19].

We hypothesized that a more solvent exposed terminal covalent analog could yield more potent BRD4 degraders and allow for a more optimal DCAF16 recruitment. Inspired by the *N*-aryl attachment handles on JQ1 used by Dragovich et al. for Genentech’s antibody-PROTAC conjugate, we sought to create *N*-aryl terminally solvent exposed covalent JQ1 analogs. We synthesized a series of terminally solvent exposed covalent analogs with an *N*-aryl linkage. The chloroacetamide (MMH249), acrylamide (MMH1), and vinyl sulfonamide (MMH2) all demonstrated a significant improvement in degradation potency. Additionally, their activity was almost entirely ablated in DCAF16 knockout (KO) K562 cells (Fig. S1). MMH249 showed a DC_50_ of 8 nM but failed to completely degrade BRD4_BD2_ and reached a D_max/16h_ of 56 %. MMH1 and MMH2, however, demonstrated nearly quantitative BRD4_BD2_ degradation (95 %), and DC_50_ values of 0.3 nM and 1 nM respectively. These compounds displayed a ~3333- and 1000-fold increase in degradation potency in comparison to GNE011.

### 2.2. Differential BRD4_BD2_ degradation potency does not correlate with a differential BRD4_BD2_ engagement

To test whether differential degradation potencies could be explained by differences in BRD4_BD2_ affinity, we measured the *in vitro* binding potencies of the covalent library against BRD4_BD1/2_ using a competition-based luminescent Amplified Luminescent Proximity Homogenous Assay (AlphaScreen). This assay utilizes donor and acceptor beads, each conjugated to a (bio)molecule of interest. Upon excitation by light, donor beads generate singlet oxygen species (^1^O_2_), that can get transferred to the acceptor beads given sufficient donor-acceptor proximity, which then emit luminescence. In this case, His tagged BRD4_BD1/2_ and biotin-tagged JQ1 (JQ1-biotin) were used in conjunction with Nickel-acceptor and Streptavidin-donor beads, respectively (Fig. 2B) [28]. The competitive displacement of JQ1-biotin by the covalent analogs was measured to determine BRD4_BD1/2_ binding potencies.

All covalent analogs tested demonstrated comparable nanomolar BRD4_BD1_ and BRD4_BD2_ binding potencies (IC_50_ values ~1–49 nM and 2–33 nM respectively; Table S1) suggesting that the differential BRD4_BD2_ degradation potencies of the covalent analogs were not due to differences in affinity for BRD4 (Fig. 2D–S6A). Likewise, all non-covalent analogs despite showing nanomolar BRD4_BD1/2_ engagement showed no significant DCAF16-depdendent BRD4_BD2_ degradation (Figs. S2, S6B, C), further suggesting that the DCAF16 covalent interaction and DCAF16-induced BRD4_BD2_ degradation have a causal relationship that is tunable by electrophilic warhead substitution.

### 2.3. Characterization of BRD4_BD2_-DCAF16 ternary complex formation induced by JQ1 analogs

**Fig. 3.**
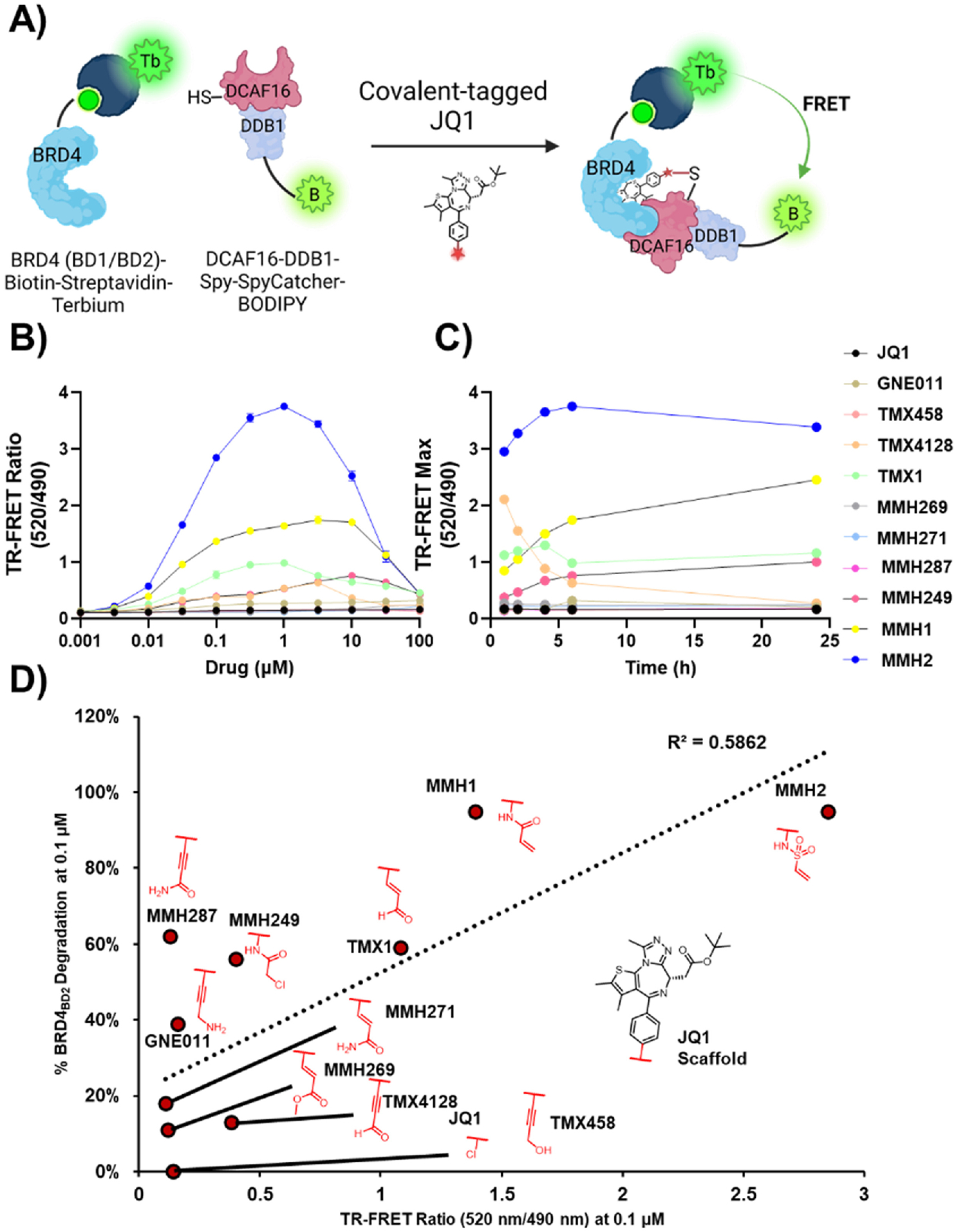
A) A schematic diagram of the BRD4_BD2_-DCAF16 TR-FRET assay. B) A 6 h TR-FRET assay for all covalent JQ1 analogs (520 nm/490 nm). C) A graph of TR-FRET maximum ratio vs. time for all covalent JQ1 analogs at 1, 2, 4, 6, and 24 h incubation times. D) A correlational analysis between the TR-FRET Ratio (520 nm/490 nm) and the % BRD4_BD2_ degradation for all JQ1 analogs.

To determine the relationship between covalent warheads and DCAF16 recruitment to BRD4_BD2_, we used a time-resolved fluorescence energy transfer (TR-FRET) assay to measure induced BRD4_BD2_-DCAF16 ternary complex formation (Fig. 3A). This assay measures fluorescence transfer from a donor fluorophore-conjugated biomolecule to an acceptor fluorophore-conjugated biomolecule, which can be facilitated by chemically induced proximity between the two biomolecules (i.e., by PROTACs or MGDs) [29]. Using biotinylated BRD4_BD2_ with terbium-tagged streptavidin (donor), and BODIPY-FL labelled DCAF16 (acceptor), TR-FRET ratio (520 nm/490 nm) measurements were made after a 6 h degrader incubation time (Fig. 3B).

Furthermore, we wanted to determine the kinetics of the BRD4_BD2_-DCAF16 interaction given its covalent nature. Hence, we conducted a time-dependent TR-FRET analysis where we measured the TR-FRET ratio after incubation for 1, 2, 4, 6, and 24 h (Fig. 3C–S4, S5). Additionally, to understand the relationship between ternary complex formation kinetics and warhead reactivity we measured the depletion of the covalent analogs in the presence of glutathione using MS (Table S2). MMH1 and MMH249 which showed modest half-lives (*t*_1/2_ = 572 and 531 min) showed a progressive ternary complex formation over time and showed the maximum TR-FRET signal at 24 h, whereas TMX1 and MMH2 (*t*_1/2_ = 415 and >750 min) showed saturation at 1 h. There did not appear to be a direct correlation between warhead reactivity and time-dependent saturation. For instance, MMH287 and TMX1, the two fastest reacting analogs (*t*_1/2_ = 380 min and 415 min), showed completely disparate ternary complex formation kinetics where unlike TMX1 (saturation at 1h) MMH287 showed almost no increase in ternary complex formation (Fig. S4). Peculiarly, TMX4128, a reactive warhead (*t*_1/2_ = 530 min) showed maximum TR-FRET signal at 1 h but showed a disintegration of the ternary complex overtime (TR-FRET_max_ = 2.12 and 0.28 at 1 and 24 h respectively).

Expectedly, non-degradative covalent analogs such as TMX458, MMH269, and MMH271, as well as the non-covalent analogs showed no significant DCAF16 recruitment (Figs. S4 and S5).

We hypothesized that the BRD4_BD2_ degradation potency of the covalent analogs would correlate with ternary complex formation (Fig. 3D). As expected, potent degraders such as TMX1, MMH1, and MMH2 showed a strong BRD4_BD2_- DCAF16 ternary complex formation with TR-FRET_max_ of 1.34, 1.78, and 3.75 respectively. However, other modest degraders such as TMX4128, MMH249, MMH287, and GNE011 demonstrated TR-FRET_max_ of 0.65, 0.77, 0.15, and 0.18, with only TMX4128 and MMH249 showing significant ternary complex formation. Non- or poorly-degrading covalent analogs such as TMX458, MMH269, and MMH271 showed minimal or no-ternary complex formation (TR-FRET_max_ = 0.16, 0.18, 0.14) on par with JQ1 (TR-FRET_max_ = 0.15; Fig. S3). To determine whether there was a linear correlation between BRD4_BD2_ degradation potency and ternary complex formation, we conducted a correlational analysis where the TR-FRET ratio was plotted against the % degradation at 0.1 μM concentration (Fig. 3D). The graph showed an R^2^ value of 0.59, indicating a modest linear trend. GNE011 and MMH287 might possibly show a disparity between their *in vitro* TR-FRET ratios and cellular BRD4 degradation since the cellular activity could be driven by a chemically reactive metabolite of the alkyne chemotype [24–27].

### 2.4. Determining cellular DCAF16-dependent BRD4 degradation potencies

**Fig. 4.**
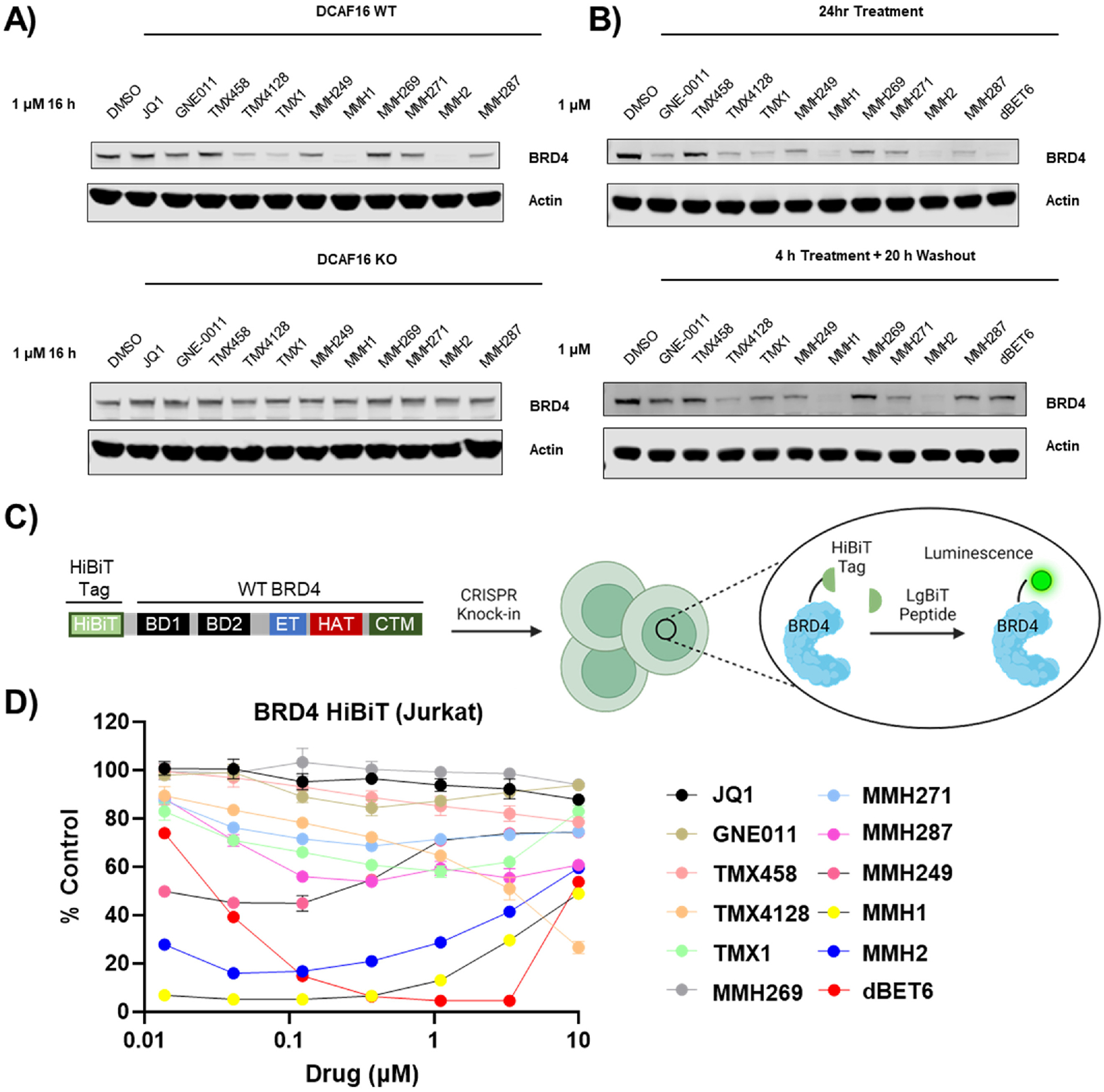
Cellular activity of the covalent BRD4 degraders. Western blotting analysis of BRD4 degradation by covalent analogs in K562 cells with A) WT and DCAF16 KO, and B) washout after 4 h of treatment (24 h total). C) A schematic depiction of the HiBiT system for quantification of protein abundance. D) Dose-dependent degradation of BRD4 by covalent JQ1 analogs in a BRD4-HiBiT JURKAT cell line. dBET6 is used as a positive control.

Western blotting was used to determine the degradation potencies of the covalent BRD4 degraders. Both wild-type and DCAF16 KO K562 cells were treated with the covalent JQ1 library at 1 μM concentrations and a 16 h incubation time (Fig. 4A). All moderate to strong degraders such as TMX1, TMX4128, MMH1, MMH2, and MMH287 showed nearly complete BRD4 degradation that was completely rescued by DCAF16 KO. Similar to the BRD4_BD2_ reporter assay results for GNE011 and MMH249 which showed a 49 and 56 % D_max_, both degraders displayed moderate degradation by Western blot (Fig. 4A).

Upon DCAF16 covalent adduct formation by covalent JQ1 analogs, the DCAF16-JQ1 covalent adduct could presumably engage in several BRD4 degradation cycles, and thereby lead to a sustained BRD4 degradation even after removal of ‘free-compound’ from the cellular media. Hence, to confirm the DCAF16-JQ1 covalent adduct formation in cells, a washout experiment was conducted. This experiment involves cell treatment and incubation with a ‘covalent’ compound, followed by a wash-step where the cells are washed and resuspended in compound-free cell media [30]. To confirm covalent-engagement of DCAF16 by covalent JQ1 degraders, K562 cells were treated with 1 μM concentrations, 4 h treatment time, followed by a washout and 20 h compound-free incubation. BRD4 degradation from the washout experiment was compared to a 24 h incubation without washout (Fig. 4B). BRD4 degradation was completely rescued by washout for the reversible cereblon-based BRD4 degrader, dBET6. TMX1, TMX4128, MMH1, MMH2, and MMH249 showed no or minimal difference in BRD4 degradation between the washout and no-washout blots. Therefore, these compounds induce BRD4 degradation via an irreversible interaction with DCAF16 commensurate with our previous findings [19]. Weaker degraders such as GNE011 and MMH287, showed nearly a complete rescue perhaps owing to the incubation time (4 h) being inadequate for weaker degraders to establish covalent DCAF16 covalent adducts. Surprisingly, MMH269 and MMH271 showed BRD4 degradation in 24 h no washout experiments suggesting these degraders might be slow reacting with DCAF16 in cells.

Thereafter, we sought to characterize the BRD4 cellular degradation potencies in a dose-dependent manner at a shorter time point to minimize secondary effects. This was performed using the bioluminescent HiBiT-BRD4 assay, which consists of an 11 amino acid peptide knock-in tag to a protein-of-interest using CRISPR insertion. A complementary polypeptide (LgBiT) detects and interacts with all HiBiT tagged proteins, thereby reconstituting a luminescent NanoBiT enzyme, that can be quantified by its bioluminescence [31]. WT and DCAF16 KO JURKAT cell lines expressing the BRD4-HiBiT system were generated to quantify dose-dependent BRD4 degradation after 6 h incubation (Fig. 4C). JQ1 and dBET6 were used as negative and positive controls respectively. Quantitative BRD4 degradation by dBET6 was maintained in both WT and DCAF16 KO cells. Only TMX1, TMX4128, MMH1, MMH2, MMH249, and MMH287 showed a noticeable BRD4 degradation that was completely rescued by DCAF16 KO (Fig. 4D–S8). Only MMH1 and MMH2 showed robust degradation (>90 % degradation at <100 nM) after 6 h, whereas modest degraders TMX1, TMX4128, and MMH249 only showed <60 % degradation. Surprisingly, TMX4128 showed ~73 % degradation albeit at 10 μM, confirming the rapid DCAF16-dependent BRD4 degradation from the washout experiments at higher concentrations.

As in the BRD4_BD2_ reporter assay, neither the non-degradative covalent nor non-covalent analogs showed any significant BRD4 degradation (Figs. S8 and S9).

## 3. Discussion

We describe the generation of a library of BRD4 degraders through addition of a minimal covalent moiety to the reversible BRD4 inhibitor JQ1. The library consists of derivatives with varying DCAF16-dependent BRD4 degradation potencies with DC_50_ values ranging from <10 nM to >10 μM. These were be achieved by subtle changes such as a transposition of the acrylamide warhead (ie., MMH1 vs MMH271). The C-aryl vs. *N*-aryl warhead linkages on the JQ1 scaffold lead to drastic changes in degradation potencies, with all *N*-aryl covalent analogs such as MMH1, MMH2, and MMH249, demonstrating the lowest DC_50_ values. This could be due to the surface presentation of the warhead to DCAF16, where *N-*aryl warheads could presumably furnish a greater solvent exposure (Fig. S7).

The high tolerance of DCAF16 for the recognition of covalent warheads on the BRD4 surface may indicate a naturally evolved function of DCAF16. Given that E3 ligases are an essential component of protein homeostasis that recognize damaged, oxidized, or dysregulated protein substates for degradation, DCAF16’s covalent sensing ability may be linked to a protein-damage or protein-oxidation response. Furthermore, DCAF16 has also been shown to endogenously ubiquitinate Spindlin 4 (SPIN4) — an epigenetic reader similar to BRD4, containing a Tudor domain which can bind trimethylated H3K4 (PDB 4UY4) — which may further indicate a natural surface complementarity with epigenetic machinery [32]. This is reminiscent of the damage/oxidation-sensing role of Von Hippel-Lindau’s (VHL) in degrading the hypoxia inducing factor-1α (HIF-1α) upon sensing a surface proline oxidation, and of CRBN’s role in removing protein fragments with C-terminal cyclized asparagine or glutamine residues [33,34].

Likewise, DCAF16-induced degradation of BRD4 upon binding covalent JQ1 analogs may mimic the native function of DCAF16 to prevent epigenetic reader-mediated transcriptional regulation by inducing the degradation of epigenetic reader proteins such as BRD4 and SPIN4, upon binding damaged histone lysine tails. Identifying post-translation modifications recognized by DCAF16 as a histone damage response, could serve as a promising chemical starting point to yield new and robust DCAF16 recruiters, similar to the retrospective success of thalidomide/lenalidomide and the discovery of the first VHL ligand [10,33,35–42].

Moreover, the *trans*-labelling covalent mechanism of these JQ1 covalent glues, where a covalent ligand reversibly binds a ligandable protein ‘A’ (i.e, BRD4), yet covalently labels another protein ‘B’ (ie., DCAF16) presents the possibility of going beyond the traditional *cis-*labelling covalent pharmacology to address ‘undruggable’ proteins.

We provide a novel potential inhibitor-to-glue conversion strategy whereby covalent tagging of preexisting inhibitors could confer a gain-of-function neointeraction which can be tuned by warhead variation.

## Supplementary Material

Multimedia component 1

Multimedia component 2

## Data Availability

Data will be made available on request.
