# Supplementary material for: Exploration of the tunability of BRD4 degradation by DCAF16 trans-labelling covalent glues
Source: Eur J Med Chem. Author manuscript; Available in PMC 2025 Apr 1. (PMC11960843; doi:10.1016/j.ejmech.2024.116904)
Supplement: Multimedia component 1 [file NIHMS2066387-supplement-Multimedia_component_1.docx]

**Supplementary Information for:**

**Exploration of the Tunability of BRD4 Degradation by DCAF16 *Trans*-labelling Covalent Glues**

Muhammad Murtaza Hassan^1,2,#^, Yen-Der Li^3,4,5,#^, Michelle W. Ma^3,6,7,#^, Mingxing Teng^8^, Woong Sub Byun^1^, Kedar Puvar^6,7^, Ryan Lumpkin^6,7^, Brittany Sandoval^4^, Justine C. Rutter^4,5^, Cyrus Y. Jin^6,7^, Michelle Y. Wang^6^, Shawn Xu^4^, Anna M. Schmoker^6,7^, Hakyung Cheong^3,6,7^, Brian J. Groendyke^6^, Jun Qi^6^, Eric S. Fischer^6,7,*^, Benjamin L. Ebert^4,5,9,*^, Nathanael S. Gray^1,*^

^1^Department of Chemical and Systems Biology, ChEM-H and Stanford Cancer Institute, Stanford School of Medicine, Stanford University, Stanford, CA

^2^SPARK Translational Research Program, Stanford University School of Medicine, Stanford, CA, USA

^3^Department of Molecular and Cellular Biology, Harvard University, Cambridge, MA

^4^Department of Medical Oncology, Dana-Farber Cancer Institute, Boston, MA

^5^Cancer Program, Broad Institute of MIT and Harvard, Cambridge, MA

^6^Department of Cancer Biology, Dana-Farber Cancer Institute, Boston, MA

^7^Department of Biological Chemistry and Molecular Pharmacology, Harvard Medical School, Boston, MA

^8^Center for Drug Discovery, Department of Pathology & Immunology, and Verna and Marrs McLean Department of Biochemistry and Molecular Pharmacology, Baylor College of Medicine, Houston, TX

^9^Howard Hughes Medical Institute, Boston, MA

^#^These authors contributed equally to this work

**Supplementary Figures and Tables**

**
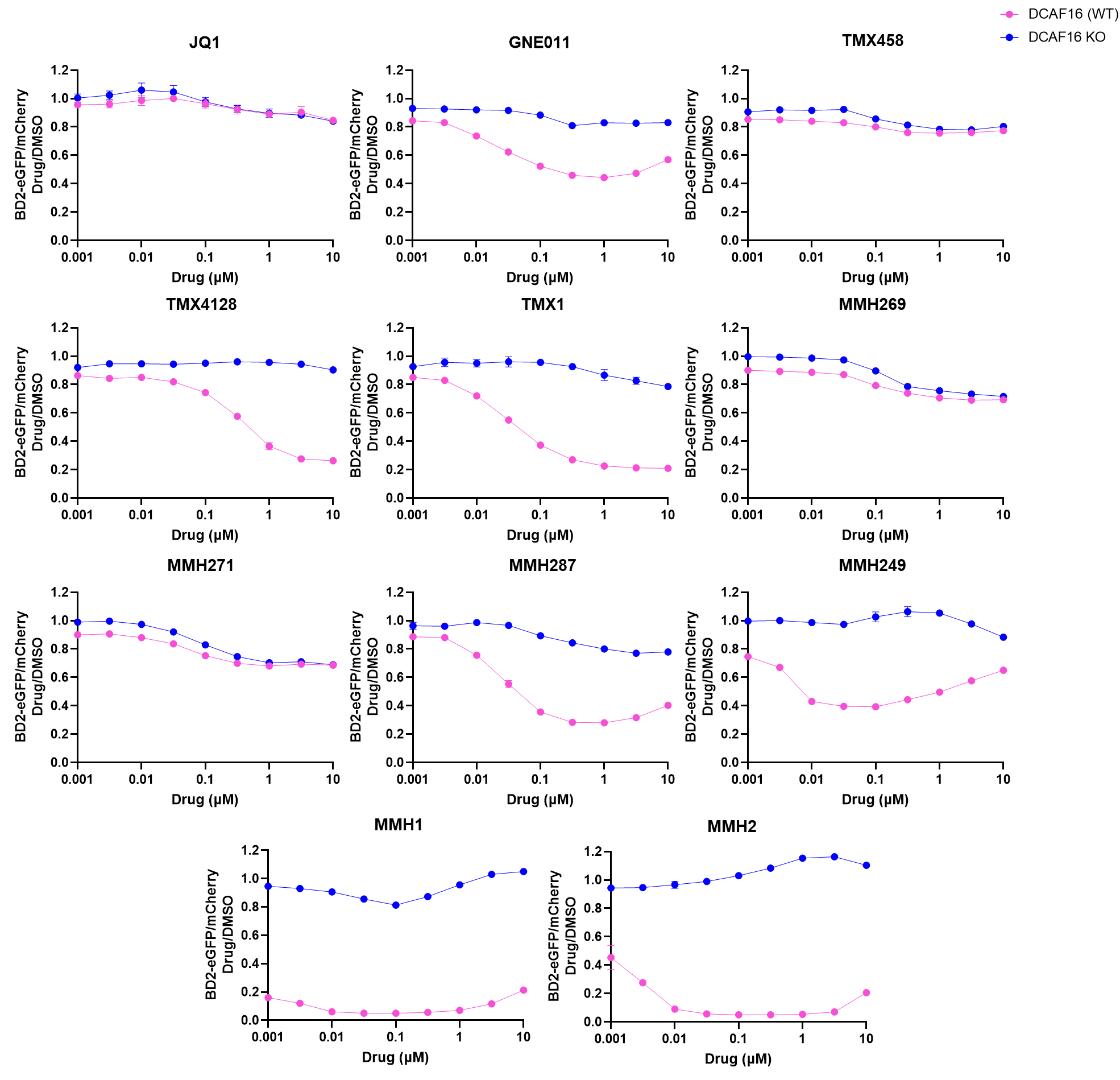
**

**Figure S1.** Degradation of BRD4_BD2_ in K562 cells (wild type vs. DCAF16 KO) via a BRD4_BD2_-eGFP and mCherry flow reporter assay for all covalent JQ1 analogs. JQ1 is included for comparison.


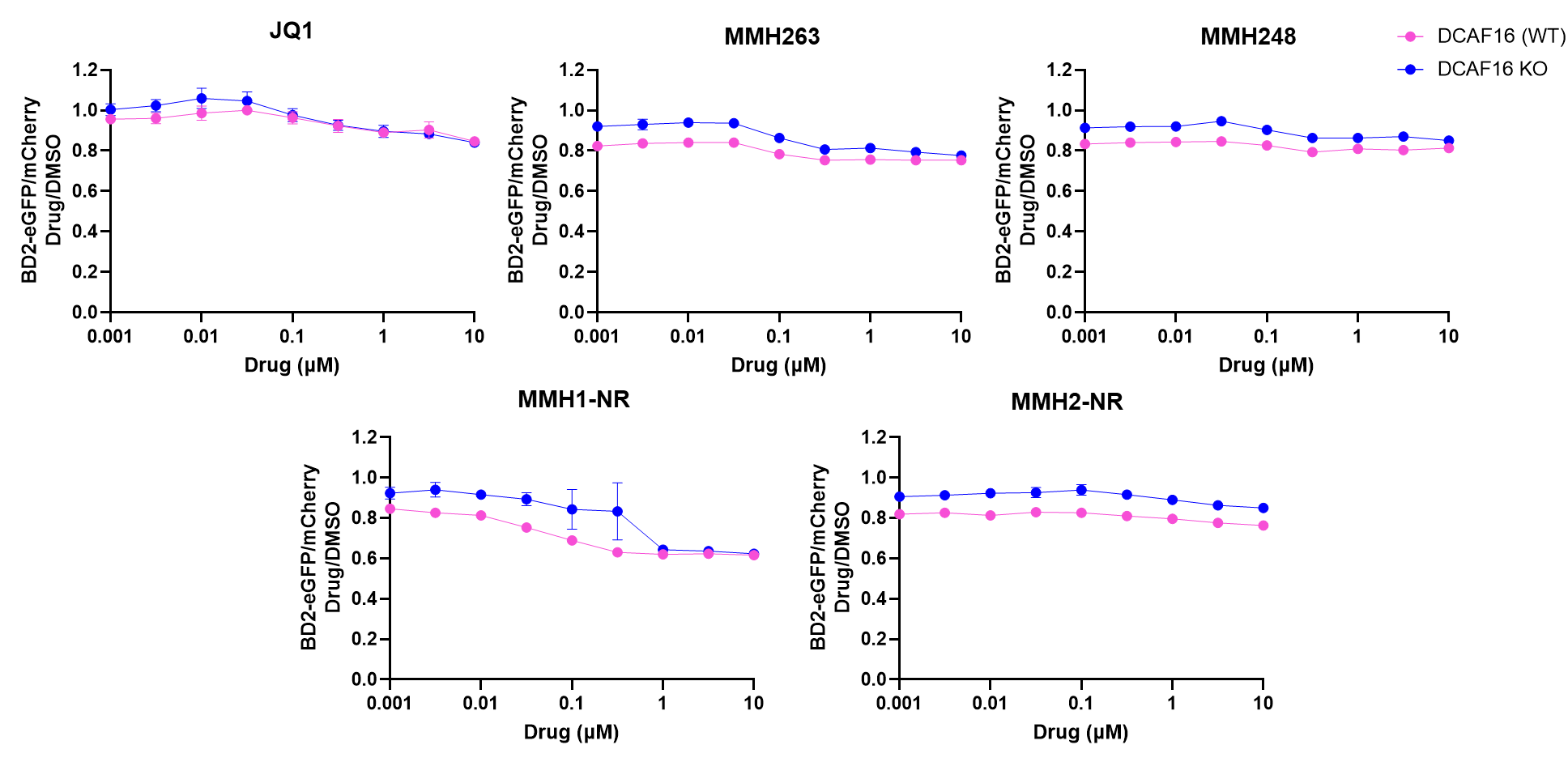


**Figure S2.** Degradation of BRD4_BD2_ in K562 cells (wild type vs. DCAF16 KO) via a BRD4_BD2_-eGFP and mCherry flow reporter assay for all non-covalent JQ1 analogs. JQ1 is included for comparison.


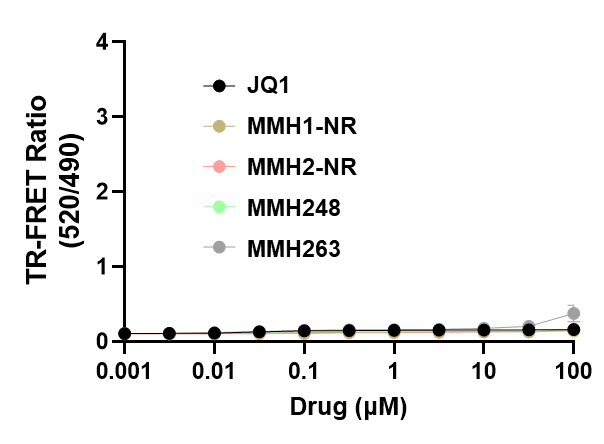


**Figure S3.** A 6 h BRD4_BD2_-DCAF16 TR-FRET assay for non-covalent analogs.

**
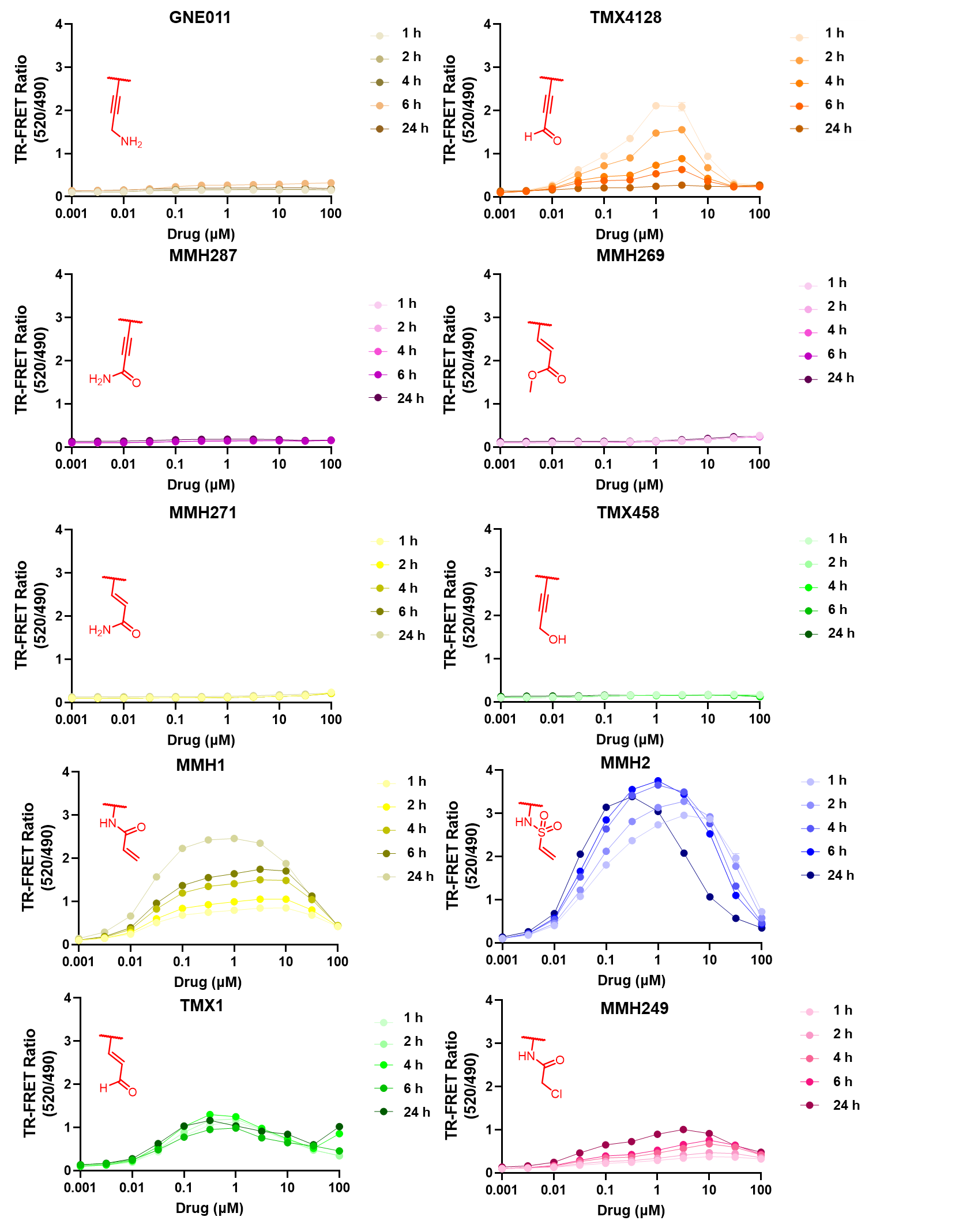
**

**Figure S4.** Time-dependent *in vitro* BRD4_BD2_-DCAF16 ternary complex formation for JQ1 covalent analogs using a TR-FRET assay. Four compound incubation times are used (1, 2, 4, 6, and 24 h) at 11 concentrations (0.001 to 100 µM). The covalent warhead appendages to the parent JQ1 scaffold are drawn in red.


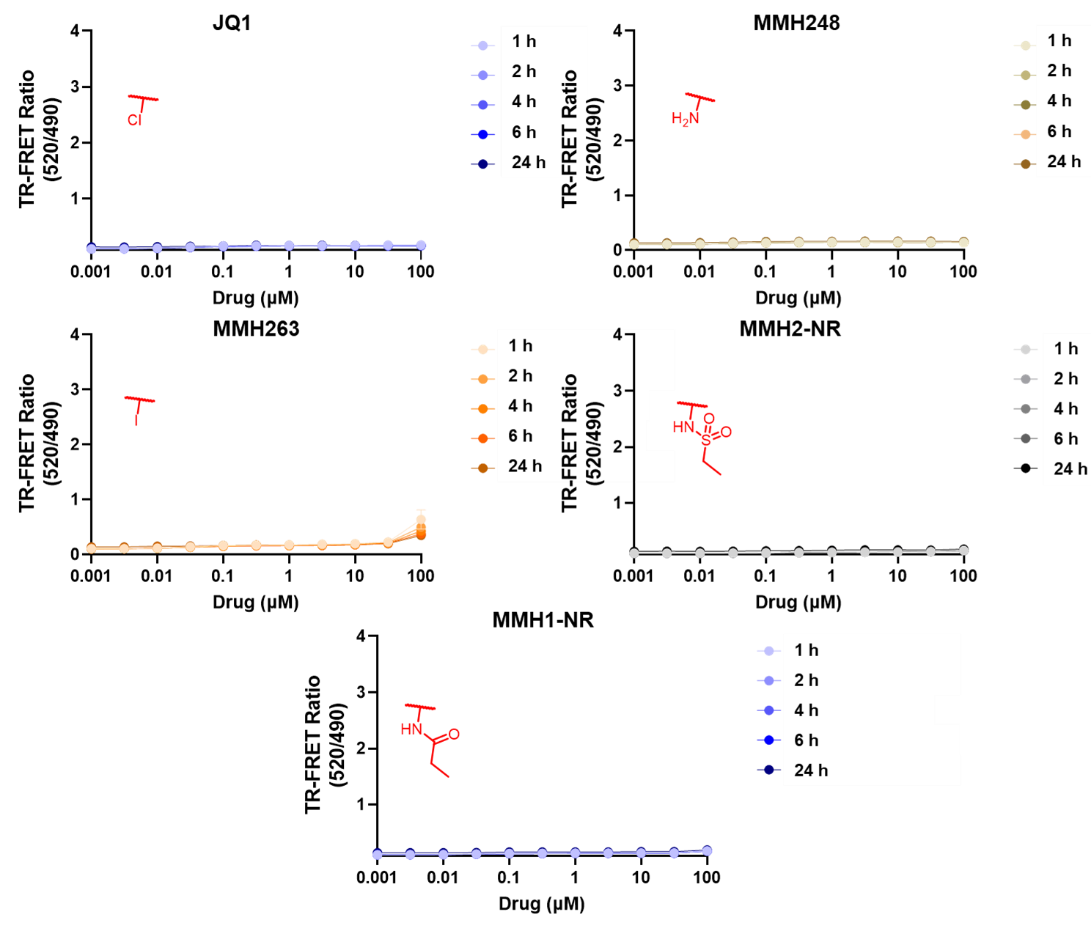


**Figure S5.** Time-dependent *in vitro* BRD4_BD2_-DCAF16 ternary complex formation for all non-covalent covalent analogs using a TR-FRET assay. Four compound incubation times are used (1, 2, 4, 6, and 24 h) at 11 concentrations (0.001 to 100 µM). The non-covalent appendages to the parent JQ1 scaffold are drawn in red.


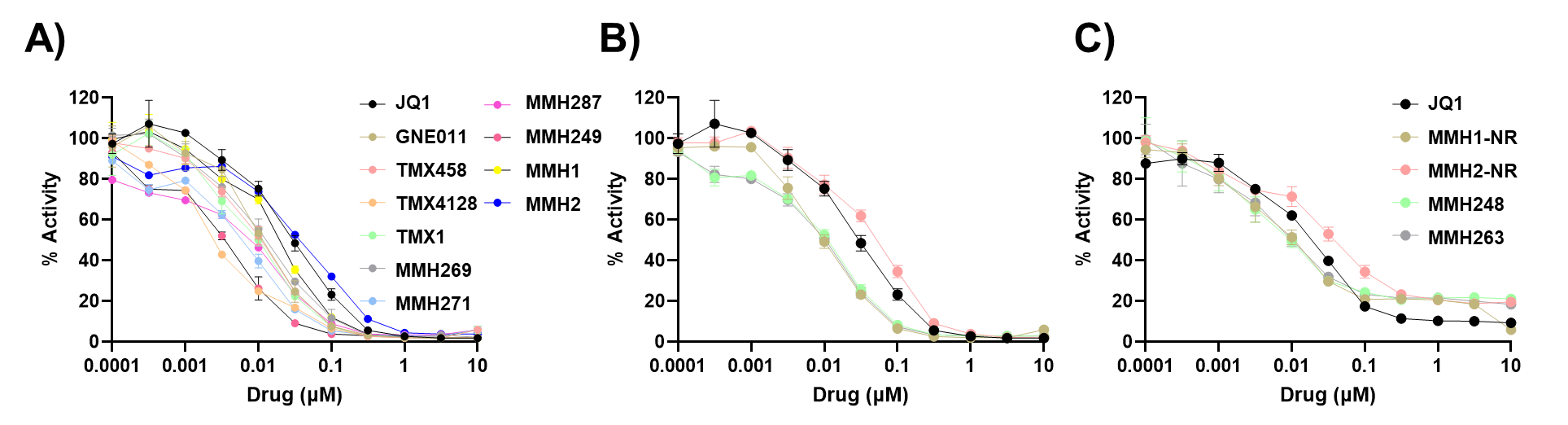


**Figure S6.** BRD4_BD1_ and BRD4_BD1_ binding potencies for JQ1 compound libraries using a competition based Alphascreen. A) BRD4_BD1_ binding potencies for covalent JQ1 analogs. B) and C) BRD4_BD1_ and BRD4_BD2_ binding potencies for non-covalent analogs.


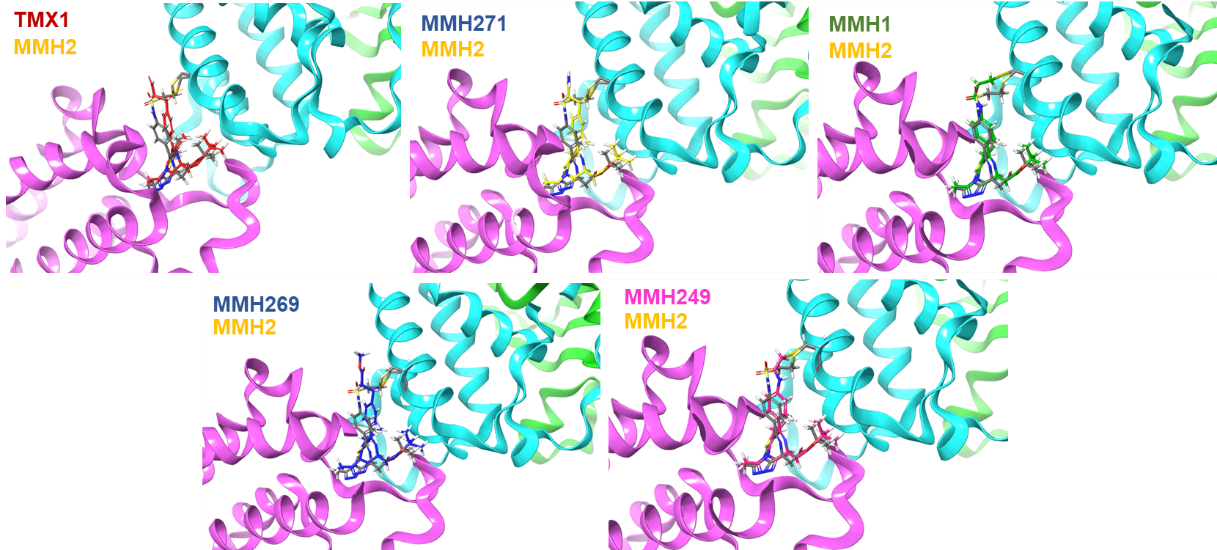


**Figure S7.** The predicted covalent docking poses of select covalent JQ1 analogs using the Schrödinger Maestro 11.9.011 software and Glide (PDB: 8G46). The docking poses of covalent JQ1 analogs are superimposed on MMH2 (yellow).


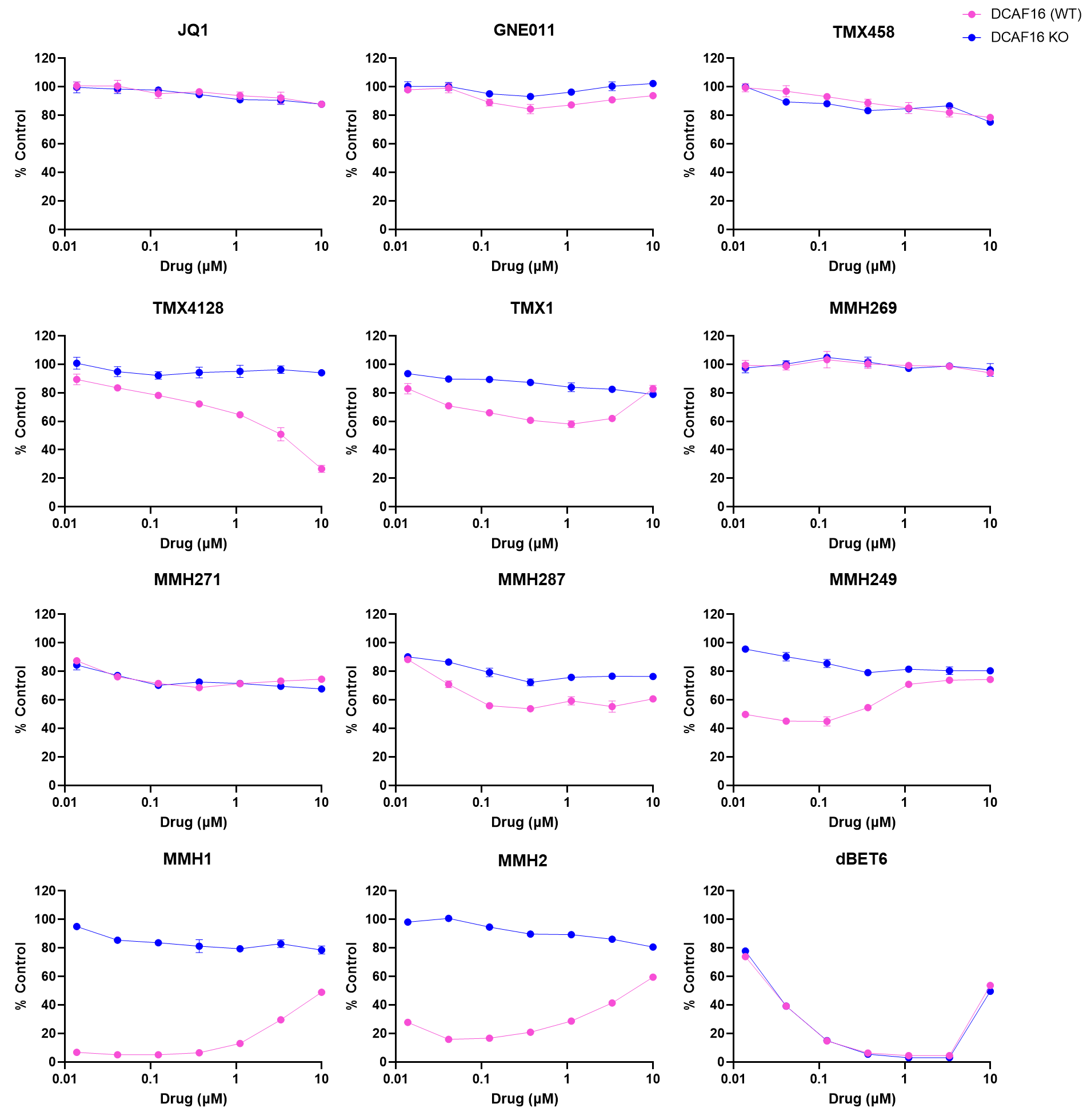


**Figure S8.** Degradation of BRD4 in JURKAT cells (wild type vs. DCAF16 KO) in a HiBiT assay (6 h) for all covalent JQ1 analogs with dBET6 as a positive control. JQ1 is included for comparison.


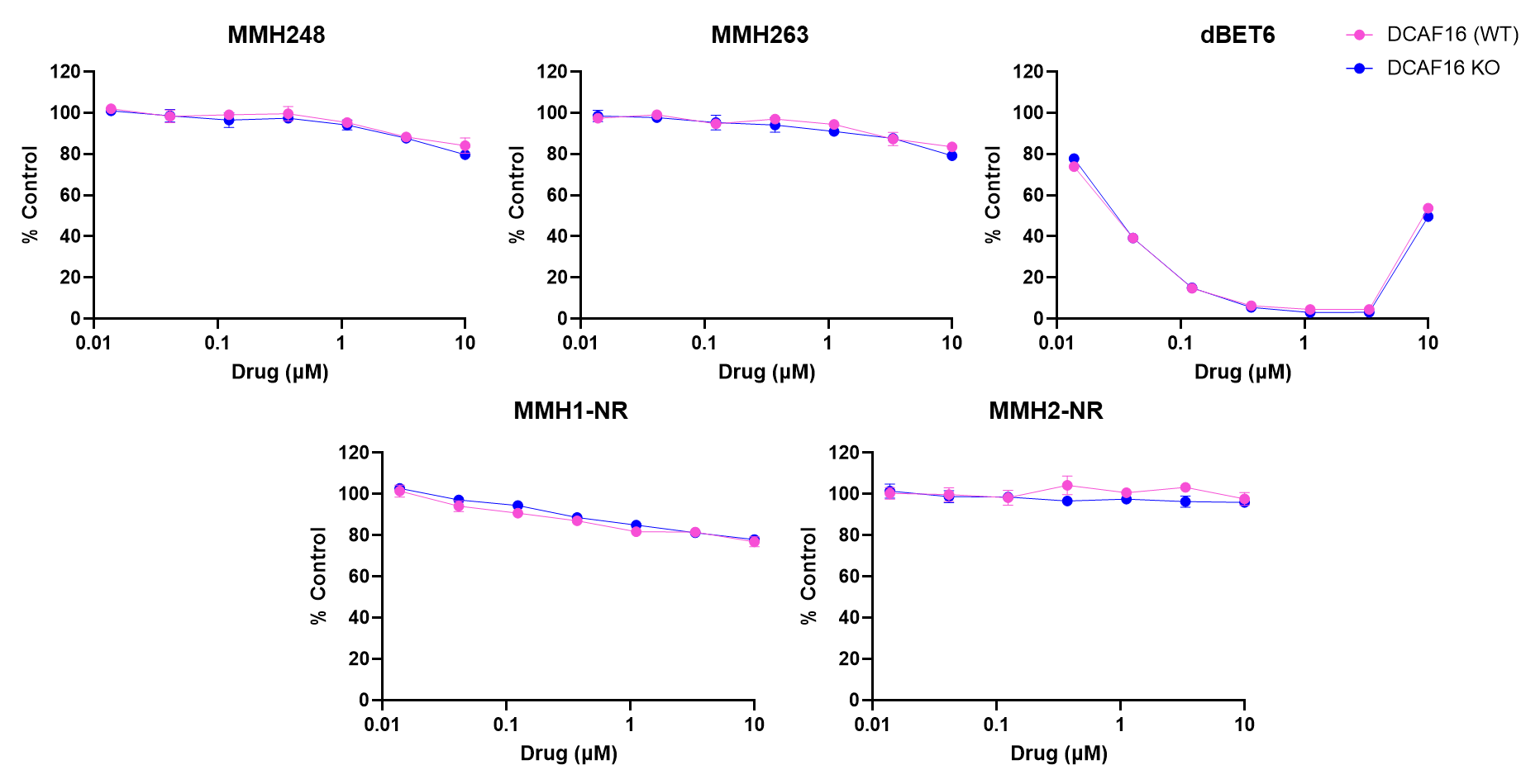


**Figure S9.** Degradation of BRD4 in JURKAT cells (wild type vs. DCAF16 KO) in a HiBiT assay (6 h) for all non-covalent JQ1 analogs, with dBET6 as a positive control.


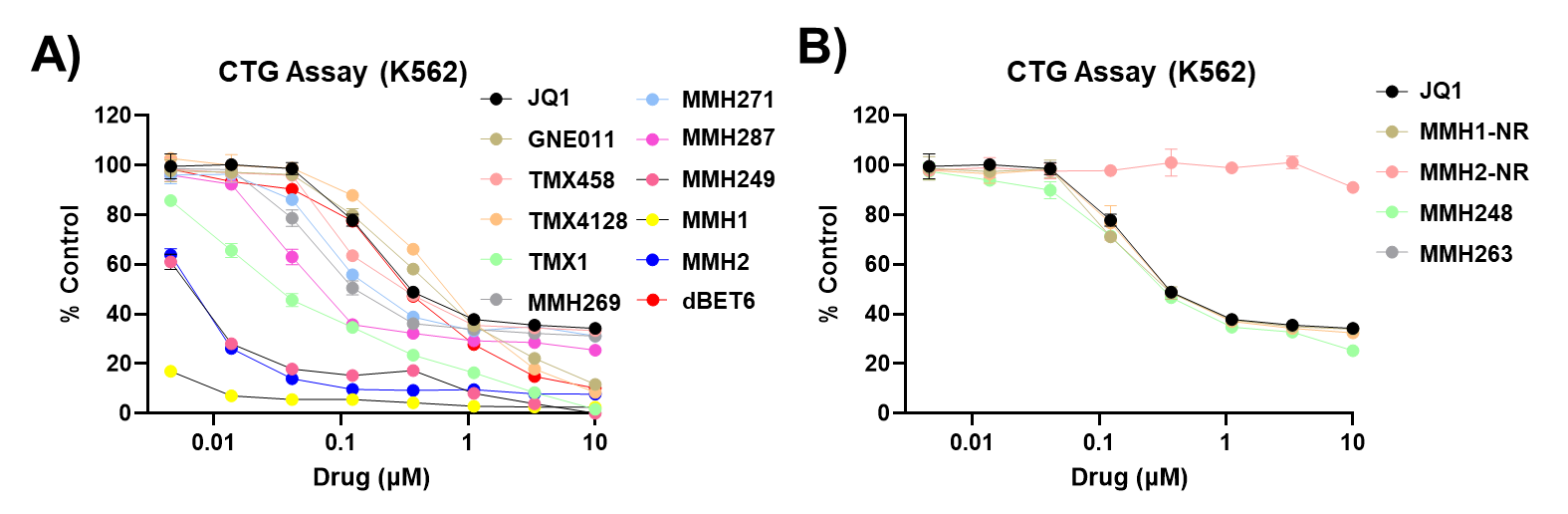


**Figure S10.** A 72 h CTG assay in K562 cells for all JQ1 analogs including both A) covalent and B) non-covalent analogs. JQ1 and dBET6 are used for comparison.

**Table S1.** IC_50_ values for all JQ1 analogs for BRD4_BD1_ and BRD4_BD2_ as determined by an AlphaScreen assay.

| **Compound** | **IC_50_ (nM)** | |
| --- | --- | --- |
|  | **BRD4_BD1_** | **BRD4_BD2_** |
| (+)-GNE-0011 | 11.16 | 7.79 |
| (+)-JQ1 | 26.99 | 18.21 |
| MMH1 | 17.67 | 15.50 |
| MMH2 | 48.60 | - |
| MMH248 | 12.49 | 4.29 |
| MMH249 | 3.98 | 2.59 |
| MMH263 | 11.66 | 4.96 |
| MMH269 | 10.54 | 5.07 |
| MMH271 | 8.08 | 7.58 |
| MMH277 | 9.72 | 6.00 |
| MMH284 | 47.18 | 20.43 |
| MMH287 | 12.91 | - |
| TMX1 | 8.78 | 33.86 |
| TMX4128 | 2.20 | 21.70 |
| TMX458 | 10.14 | 3.92 |

**Table S2.** The half-lives of JQ1 covalent analogs extrapolated from a 240 min Glutatione reactivity assay, where a >750 min half-life would result in >20% depletion in 240 min of GSH incubation with covalent analogs.

| **Glutathione Stability - Extrapolated***** | | |
| --- | --- | --- |
| Half-life (min) | | |
| Compound ID | PBS | PBS + 5 mM GSH |
|  |  |  |
| **MMH269** | > 750 | > 750 |
| **MMH271** | > 750 | > 750 |
| **MMH2** | > 750 | > 750 |
| **TMX4128** | > 750 | 530 |
| **TMX1** | > 750 | 415 |
| **GNE-011** | > 750 | 678 |
| **MMH249** | > 750 | 531 |
| **MMH1** | > 750 | 572 |
| **MMH287** | > 750 | 380 |

**Materials and Methods**

**Cell culture**

Human leukemia cell lines (JURKAT and K-562) were obtained from the American Type Culture Collection (ATCC, Manassas, VA, USA) and K562-Cas9 cell line was provided by Zuzana Tothova (Dana-Farber Cancer Institute). The cells were cultured in RPMI 1640 medium supplemented with 10% heat-inactivated FBS (Gibco, Grand Island, NY, USA), 100 units/mL penicillin, 100 µg/mL streptomycin, 0.25 µg/mL amphotericin B, and 5% glutamine. Incubation of the cells was carried out at 37 °C with 5% CO_2_ in a humidified environment. Regular mycoplasma testing was conducted using the MycoAlert mycoplasma detection kit (Lonza, Basel, Switzerland), and all cell lines tested negative for mycoplasma contamination.

**HiBiT assay**

HiBiT-edited JURKAT cells were plated in white 384-well cell culture plates (Corning #3570) at a density of 2 × 10^4^ cells per well in 50 μL of growth medium and incubated with indicated concentrations of compounds. After 6 h, the plates were subjected to Nano-Glo HiBiT Lytic detection system (Promega, #N3040) as described in manufacturer's manual. The assays were performed in biological triplicate. DC_50_ values were determined using a non-linear regression curve fit in GraphPad PRISM 9.5.1.

**Cell viability assay (CellTiter-Glo assay)**

Cell viability was evaluated using the CellTiter-Glo assay (Promega, Madison, WI, USA). Briefly, K-562 cells (1000 cells/well) were seeded in 384-well cell culture plates (Corning, #3570) and then cells were incubated with the indicated concentrations of compounds. After 72 h, the plates were subjected to CellTiter-Glo assay as described in manufacturer's manual. The proliferation assays were performed in biological triplicate. IC_50_ values were determined using a non-linear regression curve fit in GraphPad PRISM 9.5.1.

**Antibodies**

The following antibodies were used: anti-BRD4 (Bethyl Laboratories, A301-985A100), anti-β-actin (Cell Signaling Technology, #3700), anti-mouse 800CW (LI-COR Biosciences, 926-32211), anti-rabbit 680LT (LI-COR Biosciences, 925-68021).

**Commercial Compounds**

JQ1 (HY-13030), dBET6 (HY-112588) were obtained from MedChemExpress.

**Plasmids**

The following plasmids were used in this study: Cilantro (PGK.BsmBICloneSite.FlexibleLinker.eGFP.IRES.mCherry.cppt.EF1α.PuroR, Addgene 74450) for degradation characterization; sgBFP (U6.sgRNA.cppt.SFFV.tBFP) for validation of DCAF16 knockout phenotypes. The following plasmids were used for the TR-FRET assay: StrepII-Avi-DCAF16 in a pAC-derived vector, His6-3C-Spy-DDB1ΔB in a pAC-derived vector, and His6-Avi-BRD4_BD2_ in E. Coli pET100/D-TOPO vector.

**Immunoblots**

Cells were washed with PBS and lysed in RIPA lysis buffer (Thermo Fisher Scientific) with Halt Protease Inhibitor Cocktail (Thermo Fisher Scientific) and Benzonase (Sigma-Aldrich) for 20 min on ice. The insoluble fraction was removed by centrifugation, the protein concentration was quantified using a BCA protein assay kit (Thermo Fisher Scientific), and an equal amount of lysate was run on SDS–PAGE 4–12% Bis–Tris Protein Gels (Thermo Fisher Scientific) and then transferred to nitrocellulose membrane with a XCell II Blot Module Wet Tank Transfer System (Thermo Fisher Scientific). Membranes were blocked in Intercept (PBS) Blocking Buffer (LI-COR Biosciences) and incubated with primary antibodies overnight at 4 °C. The membranes were then washed in Tris-buffered saline with Tween-20 (TBS-T), incubated for 1 h with secondary IRDye-conjugated antibodies (LI-COR Biosciences) and washed three times in TBS-T for 5 min before near-infrared western blot detection on an Odyssey Imaging System (LI-COR Biosciences).

**Reporter cell line generation**

Reporter constructs were generated by BsmBI (New England Biolabs) digestion of Cilantro reporter vector and the insert containing protein of interest coding sequence, followed by ligation with T4 DNA Ligase (New England Biolabs). Constructs were transformed into Stbl3 E. coli and purified using the MiniPrep Kit (Qiagen), and sequences were confirmed by Sanger sequencing (Quintara Biosciences Service). Lentiviruses for reporters were packaged into lentivirus as follows. 0.5 × 10^6^ HEK293T cells were seeded in 2 mL of DMEM media. The next day, a packaging mix including 1.5 μg of psPAX2, 0.15 μg of pVSV-G, and 1.5 μg of transgene plasmid was prepared in 37.5 µL of OptiMEM (Thermo Fisher Scientific). This mix was combined with 9 μL of TransIT-LT1 (Mirus) and 15 µL of OptiMEM, incubated for 30 min at room temperature, and then applied dropwise to cells. Cells were allowed to incubate for another 48 h. Lentivirus was collected by 0.4 μM filters, and then transduced to 2 × 10^6^ of K562-Cas9 cells at 50% volume ratio by spin infection. One day after infection, reporter cells were selected with puromycin at a concentration of 2 μg/mL.

**DCAF16 knockout cell line generation**

sgRNAs targeting DCAF16 (sgDCAF16) or control (sgNTC) were cloned into the sgBFP vector using BsmBI cloning. In brief, vectors were linearized with BsmBI (New England Biolabs) and gel-purified with QIAquick Gel Extraction Kit (Qiagen). Annealed oligos containing sgRNA sequences were phosphorylated with T4 polynucleotide kinase (New England Biolabs) and ligated into linearized vector backbone. sgRNA constructs were transformed, purified, and verified, and lentivirus was generated as described above. Lentivirus containing sgRNA was transduced to 2 × 10^6^ of K562-Cas9 cells at 10% volume ratio by spin infection. FACS sorting was performed to enrich BFP+ cells one week after infection. For the generation of single-clone DCAF16 knockout cells, pooled K562-Cas9 cells stably expressing sgRNA targeting DCAF16 were seeded in 384-well plates at the density of 0.25 cells per well. Clonal sgDCAF16-expressing K562-Cas9 cells were isolated after one month of expansion, and the genomic sequences were validated via deep sequencing of PCR amplicons targeting sgDCAF16 cutting sites (MGH CCIB DNA Core Service).

**Flow Reporter degradation assays**

K562 cells stably expressing degradation reporter were dosed with DMSO or degraders at various times and concentrations using D300e Digital Dispenser (HP). The fluorescent signal was quantified by flow cytometry (LSRFortessa flow cytometer, BD Biosciences) and analyzed using FlowJo (flow cytometry analysis software, BD Biosciences). The geometric mean of the eGFP and mCherry fluorescent signal for round and mCherry-positive cells was calculated. GFP expression was normalized to mCherry signal and drug treatments were compared to DMSO controls.

***In silico* Modelling**

The cryo-EM structure of MMH2 induced BRD4_BD2_-DCAF16-DDB1 ternary structure (PDB 8G46) was used for pose-prediction of the covalent analogs. Default settings for ligand preparation, and protein preparation and refinement were used. Cys58 was selected as the reactive residue. The centroid of the workspace ligand was selected to be that of MMH2. The Michael addition reaction type was chosen, with a ‘pose-prediction’ Docking Mode. The output poses per ligand was restricted to 5.

**Intact protein mass spectrometry**

Prior to intact mass analysis, recombinant human DDB1ΔB-DCAF16 variants were incubated with DMSO, TMX1, KB02-JQ1, MMH1, or MMH2 with and without the presence of recombinant human BRD4BD2 for 16 h at 4°C. For GNE11, recombinant proteins were incubated with drug at room temperature for 16 h. Intact mass analysis of DCAF16 variants was performed similarly to a previously described protocol49 with modifications. Briefly, drug-treated proteins were injected on a self-packed column (6 cm POROS 50R2 packed in 0.5 mm I.D. tubing), desalted for 4 minutes, and then eluted to an LTQ ion trap mass spectrometer (Thermo Fisher Scientific) using an HPLC gradient (0-100% B in 20 minutes, A=0.1M acetic acid, B=0.1 M acetic acid in acetonitrile, ESI spray voltage=5kV). The mass spectrometer acquired full scan mass spectra (m/z 300-2000) in profile mode. Mass spectra were deconvoluted using MagTran version 1.03 b250. Labeling efficiency was calculated from zero charge mass spectra using peak heights according to [peak height labeled protein] / [peak height labeled protein + peak height unlabeled protein] x 100%.

**DDB1-DCAF16–BRD4_BD2_ TR-FRET**

Titrations of compounds to induce the DCAF16-BRD4BD complex were carried out by mixing 100 nM biotinylated BRD4_BD2_, 500 nM BODIPY-FL labeled DDB1ΔB-DCAF16 variants, and 2 nM terbium-coupled streptavidin (Invitrogen) in an assay buffer containing 50 mM HEPES pH 8.0, 200 mM NaCl, 0.1% Pluronic F-68 solution (Sigma), 0.5% bovine serum albumin (BSA) (w/v) and 1 mM TCEP. After dispensing the assay mixture (15 μL volume), increasing concentrations of compounds were dispensed in a 384-well microplate (Corning, 4514) using a D300e Digital Dispenser (HP) normalized to 1% DMSO. After excitation of terbium fluorescence at 337 nm, emission at 490 nm (terbium) and 520 nm (BODIPY FL) were recorded with a 70 μs delay over 600 μs to reduce background fluorescence, and the reaction was followed over 60 cycles of each data point using a PHERAstar FS microplate reader (BMG Labtech). The TR-FRET signal of each data point was extracted by calculating the 520/490 nm ratio.

**GSH Stability Assay**

Individual compound spiking solutions were prepared at 100 µM in DMSO and spiked into glass HPLC autosampler vials containing either phosphate buffered saline (PBS) or PBS containing 5 mM reduced glutathione. The samples were immediately injected onto an LC-MS/MS to determine reaction at time zero. At specific timepoints over four hours, the samples were reinjected to determine compound depletion.

**BRD4 AlphaScreen**

Assays were performed with minimal modifications from the manufacturer’s protocol (PerkinElmer, USA). All reagents were diluted in 50 mM HEPES, 150 mM NaCl, 0.1% w/v BSA, 0.01% w/v Tween20, pH 7.5, and allowed to equilibrate to room temperature prior to addition to plates. After addition of Alpha beads to master solutions, all subsequent steps were performed under low light conditions. A 2x solution of components with final concentrations of His-BRD4-BD1 or His-BRD4-BD2 at 40 nM, Ni-coated Acceptor Bead at 15 µg/ml, and biotinylated-JQ1 at 20nM was added in 10 µL to 384-well plates (AlphaPlate-384, PerkinElmer, USA). Plates were spun down at 150x g, and 100 nL of compound in DMSO from stock plates were added by pin transfer using a Janus Workstation (PerkinElmer, USA). Streptavidin-coated donor beads (15 µg/ml final concentration) were added as to the solution in a 2x, 10 µL volume. Following this addition, plates were sealed with foil to prevent light exposure and evaporation. The plates were spun down again at 150g. Plates were incubated at room temperature for 1 hour, and then read on an Envision 2104 (PerkinElmer, USA), using the manufacturer’s protocol.
