# Supplementary material for: Exploration of the tunability of BRD4 degradation by DCAF16 trans-labelling covalent glues
Source: Eur J Med Chem. Author manuscript; Available in PMC 2025 Apr 1. (PMC11960843; doi:10.1016/j.ejmech.2024.116904)
Supplement: Multimedia component 2 [file NIHMS2066387-supplement-Multimedia_component_2.docx]

**Synthetic Methods for:**

**Exploration of the Tunability of BRD4 Degradation by DCAF16 *Trans*-labelling Covalent Glues**

Muhammad Murtaza Hassan^1,2,#^, Yen-Der Li^3,4,5,#^, Michelle W. Ma^3,6,7,#^, Mingxing Teng^8^, Woong Sub Byun^1^, Kedar Puvar^6,7^, Ryan Lumpkin^6,7^, Brittany Sandoval^4^, Justine C. Rutter^4,5^, Cyrus Y. Jin^6,7^, Michelle Y. Wang^6^, Shawn Xu^4^, Anna M. Schmoker^6,7^, Hakyung Cheong^3,6,7^, Brian J. Groendyke^6^, Jun Qi^6^, Eric S. Fischer^6,7,*^, Benjamin L. Ebert^4,5,9,*^, Nathanael S. Gray^1,*^

^1^Department of Chemical and Systems Biology, ChEM-H and Stanford Cancer Institute, Stanford School of Medicine, Stanford University, Stanford, CA

^2^SPARK Translational Research Program, Stanford University School of Medicine, Stanford, CA, USA

^3^Department of Molecular and Cellular Biology, Harvard University, Cambridge, MA

^4^Department of Medical Oncology, Dana-Farber Cancer Institute, Boston, MA

^5^Cancer Program, Broad Institute of MIT and Harvard, Cambridge, MA

^6^Department of Cancer Biology, Dana-Farber Cancer Institute, Boston, MA

^7^Department of Biological Chemistry and Molecular Pharmacology, Harvard Medical School, Boston, MA

^8^Center for Drug Discovery, Department of Pathology & Immunology, and Verna and Marrs McLean Department of Biochemistry and Molecular Pharmacology, Baylor College of Medicine, Houston, TX

^9^Howard Hughes Medical Institute, Boston, MA

^#^These authors contributed equally to this work

**Chemical Procedures**

**General Procedure**

All solvents and chemical reagents were purchased from commercial vendors such as Sigma Aldrich, Oakwood Chemicals, Fischer Scientific, Combi Blocks, TCI Chemicals, and Alfa Aesar. Anhydrous solvents such as THF, and DMF were used from Sure-Seal bottles without further drying treatments. All reactions were conducted in a scintillation vial and were monitored by LCMS. Compound purity was determined by the relative area of compound peaks as determined by the automatic integration of the UV spectra.

**Analytical Conditions and Instrumentation**

All NMR spectra were obtained on a 500 MHz Bruker Avance III spectrometer or on 500 MHz Bruker Avance Neo equipped with carbon detect, liquid nitrogen cooled, Prodigy cryoprobe. All deuterated solvents were purchased from either Sigma Aldrich or Cambridge isotope laboratories. Coupling constants (*J*) are reported in Hertz (Hz) and multiplicities are reported as singlet (s), doublet (d), triplet (t), quartet (q), pentet (p), sextet (sex), septet (sep), multiplet (m), and broad (br), and the combination of these are listed as a combination of their abbreviations.

LCMS was conducted on an Acquity I-Class LCMS system equipped with a PDA-UV system, a QDa mass detector, and a dual column set-up (BEH and CSH columns). The solvent gradient consisted of LCMS grade acetonitrile purchased from Fischer Scientific, and milliQ water. A gradient of 10 to 100% over 2.5 minutes followed by a 0.5 minute flush, was used for the final compounds described herein.

Preparative HPLC was used to purify all final compounds unless otherwise stated. HPLC grade methanol purchased from Fischer Scientific, and milliQ water, both containing 0.045% trifluoroacetic acid (TFA) were used for the solvent system. A 254 nm UV-light absorption was used to visualize compound chromatograms. Two methods were used for the solvent gradient system:

Method 1: Waters Sunfire C18 column (19 mm X 50 mm, 5 μm) using a gradient of 15-95% methanol in water over 60 min at a flow rate of 43 mL/min.

Method 2: Waters Sunfire C18 column (30 mm X 250 mm, 5 µm) using a gradient of 10 to 100% methanol in water containing 0.045% TFA over 40 min followed by a 5 min flush with 100% methanol, at a flow rate of 40 mL/min.

**Abbreviations**

B_2_pin_2_, bis(pinacolato)diboron; Pd_2_dba_3_, tris(dibenzylideneacetone)dipalladium(0); XPhos, dicyclohexyl[2′,4′,6′-tris(propan-2-yl)[1,1′-biphenyl]-2-yl]phosphane; KOAc, potassium acetate; NaI, sodium iodide; Chloramine-T, sodium chloro(4-methylbenzene-1-sulfonyl)azanide; THF, tetrahydrofuran; PdCl_2_(PPh_3_)_2_, bis(triphenylphosphine)palladium(II) dichloride; Et_3_N, triethylamine; DMF, dimethylformamide; rt, room temperature; K_2_CO_3_, potassium carbonate; KCl, potassium chloride; [n-Bu_4_N]OAc, tetrabutylammonium acetate; Pd(OAc)_2_, palladium(II) acetate; Xphos-PdG2, Chloro(2-dicyclohexylphosphino-2′,4′,6′-triisopropyl-1,1′-biphenyl)[2-(2′-amino-1,1′-biphenyl)]palladium(II); DCM, dichloromethane; DIPEA, diisopropylethylamine; prep, preparative; LC, liquid chromatography; MS, mass spectrometry.

**Synthetic Scheme 1. Synthesis of Aryl-linked JQ1 Analogs.**

**Synthetic Scheme 2. Synthesis of N-Aryl-linked JQ1 Analogs.**

**Chemical Synthesis and Compound Characterization**

**tert-butyl (S)-2-(2,3,9-trimethyl-4-(4-(4,4,5,5-tetramethyl-1,3,2-dioxaborolan-2-yl)phenyl)-6H-thieno[3,2-f][1,2,4]triazolo[4,3-a][1,4]diazepin-6-yl)acetate (Int-1)**

To a solution of (+)-JQ-1 (600.0 mg, 1.31 mmol) and B_2_pin_2_ (332.0 mg, 1.31 mmol) in 1,4-dixoane (5.0 mL) was added XPhos (31.0 mg, 0.065 mmol), Pd_2_dba_3_ (30.0 mg, 0.033 mmol), and KOAc (386 mg, 3.93 mmol). The reaction mixture was stirred at 100 ^o^C for 17 hours. The reaction mixture was purified via column chromatography (silica gel, eluted with 0% to 15% methanol in dichloromethane) to give **Int-1** (395 mg, 64% yield) as a yellow oil. For **Int-1**, MS (ESI) for C_29_H_38_BN_4_O_4_S [M+H]^+^: m/z calcd, 549.51; found, 549.49.

**tert-butyl (S)-2-(4-(4-iodophenyl)-2,3,9-trimethyl-6H-thieno[3,2-f][1,2,4]triazolo[4,3-a][1,4]diazepin-6-yl)acetate (MMH263)**

To a solution of **Int-1** (200.0 mg, 0.43 mmol) in 1,4-dixoane/H_2_O (v/v=2/1, 3.0 mL) was added NaI (129.0 mg, 0.86 mmol) and Chloramine-T 294.0 mg, 1.29 mmol). The reaction mixture was stirred at 50 ^o^C for 4 hours. The reaction mixture was purified directly via prep HPLC (method 1) to give **MMH263** (142 mg, 60% yield) as a yellow solid. ^1^H NMR (500 MHz, DMSO-*d*_6_) δ 7.81 (d, *J* = 8.5 Hz, 2H), 7.20 (d, *J* = 8.0 Hz, 2H), 4.40 (dd, *J* = 8.0, 6.5 Hz, 1H), 3.35-3.29 (m, 2H), 2.60 (s, 3H), 2.41 (s, 3H), 1.63 (s, 3H), 1.42 (s, 9H). MS (ESI) for C_23_H_26_IN_4_O_2_S [M+H]^+^: m/z calcd, 549.45; found, 549.38.

**tert-butyl (S)-2-(4-(4-(3-((tert-butoxycarbonyl)amino)prop-1-yn-1-yl)phenyl)-2,3,9-trimethyl-6H-thieno[3,2-f][1,2,4]triazolo[4,3-a][1,4]diazepin-6-yl)acetate (Int-2)**

To a solution of **MMH263** (40.0 mg, 0.073 mmol) and N-Boc-propargylamine (23.0 mg, 0.15 mmol) in Et_3_N/DMF (v/v=1/1, 1.2 mL) was added PdCl_2_(PPh_3_)_2_ (5.1 mg, 7.3 µmol), and CuI (2.8 mg, 14.6 µmol). The reaction mixture was stirred at rt for 3 hours. The reaction mixture was purified directly via prep HPLC (method 1) to give **Int-2** (33.1 mg, 79% yield) as a yellow oil. MS (ESI) for C_31_H_38_N_5_O_4_S [M+H]^+^: m/z calcd, 576.26; found, 576.18.

**tert-butyl (S,E)-2-(2,3,9-trimethyl-4-(4-(3-oxoprop-1-en-1-yl)phenyl)-6H-thieno[3,2-f][1,2,4]triazolo[4,3-a][1,4]diazepin-6-yl)acetate (TMX1)**

To a solution of **MMH263** (35.0 mg, 0.064 mmol) and 3,3-diethoxyprop-1-ene (13.0 mg, 0.096 mmol) in DMF (0.6 mL) was added K_2_CO_3_ (18.0 mg, 0.13 mmol), KCl (5.0 mg, 0.064 mmol), [n-Bu_4_N]OAc (39.0 mg, 0.13 mmol), and Pd(OAc)_2_ (4.0 mg, 0.019 mmol). The reaction mixture was stirred at 90 ^o^C for 8 hours. The reaction mixture was purified directly via prep HPLC (method 1) to give **TMX1** (15.3 mg, 50% yield) as a yellow oil. ^1^H NMR (500 MHz, DMSO-*d*_6_) δ 9.69 (d, *J* = 7.5 Hz, 1H), 7.81 (d, *J* = 8.5 Hz, 2H), 7.76 (d, *J* = 16.5 Hz, 1H), 7.49 (d, *J* = 8.0 Hz, 2H), 6.91 (dd, *J* = 16.0, 7.5 Hz, 1H), 4.45 (dd, *J* = 8.0, 6.5 Hz, 1H), 3.40-3.29 (m, 2H), 2.61 (s, 3H), 2.42 (s, 3H), 1.64 (s, 3H), 1.44 (s, 9H). MS (ESI) for C_26_H_29_N_4_O_3_S [M+H]^+^: m/z calcd, 477.20; found, 477.50.

**tert-butyl (S)-2-(4-(4-(3-aminoprop-1-yn-1-yl)phenyl)-2,3,9-trimethyl-6H-thieno[3,2-f][1,2,4]triazolo[4,3-a][1,4]diazepin-6-yl)acetate ((S)-GNE-0011)**

A solution of **Int-2** (45.0 mg, 0.078 mmol) in *t*-BuOH/4.0 M HCl solution in 1,4-dixoane (v/v=1/1, 1.0 mL) was stirred at rt for 1.5 hours. The reaction mixture was purified directly via prep HPLC to give **(*S*)-GNE-0011 as a TFA salt** (20.0 mg, 41% yield) as a yellow oil. ^1^H NMR (500 MHz, DMSO-*d*_6_) δ 8.35 (brs, 3H), 7.53 (d, *J* = 8.5 Hz, 2H), 7.47 (d, *J* = 8.3 Hz, 2H), 4.45 (dd, *J* = 8.2, 6.2 Hz, 1H), 4.02 (q, *J* = 5.5 Hz, 2H), 3.39-3.28 (m, 2H), 2.61 (s, 3H), 2.42 (s, 3H), 1.63 (s, 3H), 1.43 (s, 9H). MS (ESI) for C_26_H_30_N_5_O_2_S [M+H]^+^: m/z calcd, 476.21; found, 476.31.

**tert-butyl (S)-2-(4-(4-(3-hydroxyprop-1-yn-1-yl)phenyl)-2,3,9-trimethyl-6H-thieno[3,2-f][1,2,4]triazolo[4,3-a][1,4]diazepin-6-yl)acetate (TMX458)**

To a solution of **MMH263** (20.0 mg, 0.037 mmol) and propargyl alcohol (2.04 mg, 0.037 mmol) in Et_3_N/DMF (v/v=0.5 µL/0.5 mL) was added PdCl_2_(PPh_3_)_2_ (5.0 mg, 7.3 µmol), and CuI (2.0 mg, 10.4 µmol). The reaction mixture was stirred at rt for 3 hours and subsequently purified by HPLC (method 1) to yield **TMX458** (10.0 mg, 57.6% yield). ^1^H NMR (500 MHz, DMSO) δ 7.48 (d, *J* = 8.6 Hz, 2H), 7.42 (d, *J* = 8.2 Hz, 2H), 4.44 (dd, *J* = 8.2, 6.2 Hz, 1H), 4.31 (s, 2H), 3.42 – 3.26 (m, 2H), 2.61 (s, 3H), 2.43 (s, 3H), 1.64 (s, 3H), 1.43 (s, 9H). MS (ESI) for C_26_H_28_N_4_O_3_S [M+H]^+^: m/z calcd, 477.60; found 477.31.

**tert-butyl (S)-2-(2,3,9-trimethyl-4-(4-(3-oxoprop-1-yn-1-yl)phenyl)-6H-thieno[3,2-f][1,2,4]triazolo[4,3-a][1,4]diazepin-6-yl)acetate (TMX4128)**

To a solution of **MMH263** (8.0 mg, 0.015 mmol) and propiolaldehyde (0.82 mg, 0.015 mmol) in Et_3_N/DMF (v/v=0.5 µL/0.5 mL) was added PdCl_2_(PPh_3_)_2_ (2.0 mg, 2.9 µmol), and CuI (0.8 mg, 4.2 µmol). The reaction mixture was stirred at rt for 3 hours and subsequently purified by HPLC (method 1) to yield **TMX4128** (3.6 mg, 50.5% yield). ^1^H NMR (500 MHz, DMSO) δ 9.45 (s, 1H), 7.76 (d, *J* = 8.2 Hz, 2H), 7.53 (d, *J* = 8.1 Hz, 2H), 4.47 (dd, *J* = 8.1, 6.2 Hz, 1H), 3.46 – 3.27 (m, 2H), 2.61 (s, 3H), 2.43 (s, 3H), 1.64 (s, 3H), 1.44 (s, 9H). MS (ESI) for C_26_H_26_N_4_O_3_S [M+H]^+^: m/z calcd, 475.58; found 475.26.

**tert-butyl (S)-2-(4-(4-(3-amino-3-oxoprop-1-yn-1-yl)phenyl)-2,3,9-trimethyl-6H-thieno[3,2-f][1,2,4]triazolo[4,3-a][1,4]diazepin-6-yl)acetate (MMH287)**

To a solution of **MMH263** (30.0 mg, 0.055 mmol) and propiolamide (7.56 mg, 0.11 mmol) in Et_3_N/DMF (v/v=1/1, 1.2 mL) was added PdCl_2_(PPh_3_)_2_ (15.4 mg, 0.022 mmol), and CuI (6.25 mg, 0.033 mmol). The reaction mixture was stirred at rt for 4 hours. The reaction mixture was purified twice via prep HPLC (method 2) to give **MMH287** (6 mg, 23% yield). ^1^H NMR (500 MHz, DMSO) δ 8.19 (br, 1H), 7.72 (br, 1H), 7.62 (d, *J* = 8.19 Hz, 2H), 7.49 (d, *J* = 8.06 Hz, 2H), 4.45 (t, J = 7.2 Hz, 1H), 3.40-3.29 (m, 2H), 2.61 (s, 3H), 2.43 (s, 3H), 1.63 (s, 3H), 1.43 (s, 9H). MS (ESI) for C_26_H_27_N_5_O_3_S [M+H]^+^: m/z calcd, 490.60; found 490.37.

**methyl (S,E)-3-(4-(6-(2-(tert-butoxy)-2-oxoethyl)-2,3,9-trimethyl-6H-thieno[3,2-f][1,2,4]triazolo[4,3-a][1,4]diazepin-4-yl)phenyl)acrylate (MMH269)**

To a solution of **MMH263** (20.9 mg, 0.03 mmol) in 3 mL of toluene, was added cesium carbonate (37.2 mg, 0.114 mmol), Xphos Pd G2 (5.08 mg, 0.0065 mmol), and methyl acrylate (3.6 mg, 0.0418). The reaction was heated to 110 ^o^C and left to stir overnight. The reaction was filtered through celite and evaporated under reduced pressure. The mixture was purified by HPLC (method 2) to yield **MMH269** (8.91 mg, 46.3% yield). ^1^H NMR (500 MHz, DMSO) δ 7.79 (d, *J* = 8.3 Hz, 2H), 7.68 (d, *J* = 16.1 Hz, 1H), 7.46 (d, *J* = 8.0 Hz, 2H), 6.72 (d, *J* = 16.1 Hz, 1H), 4.45 (ddd, *J* = 7.7, 6.2, 1.4 Hz, 1H), 3.74 (s, 3H), 3.39 – 3.29 (m, 2H), 2.62 (s, 3H), 2.43 (d, *J* = 0.9 Hz, 3H), 1.64 (d, *J* = 0.9 Hz, 3H), 1.44 (s, 9H). MS (ESI) for C_27_H_30_N_4_O_4_S [M+H]^+^: m/z calcd, 507.63; found 507.31.

**tert-butyl (S,E)-2-(4-(4-(3-amino-3-oxoprop-1-en-1-yl)phenyl)-2,3,9-trimethyl-6H-thieno[3,2-f][1,2,4]triazolo[4,3-a][1,4]diazepin-6-yl)acetate (MMH271)**

To a solution of **MMH263** (12.0 mg, 0.022 mmol) in 3 mL of toluene, was added cesium carbonate (21.4 mg, 0.066 mmol), Xphos Pd G2 (4.73 mg, 0.006 mmol), and acrylamide (3.34 mg, 0.047). The reaction was heated to 110 ^o^C and left to stir overnight. The reaction was filtered through celite and evaporated under reduced pressure. The mixture was purified by HPLC (method 2) to yield **MMH271** (3.48 mg, 32.2% yield).^1^H NMR (500 MHz, MeOD) δ 7.64 (d, *J* = 8.5 Hz, 2H), 7.58 (d, *J* = 15.9 Hz, 1H), 7.52 (d, *J* = 8.4 Hz, 2H), 6.73 (d, *J* = 15.8 Hz, 1H), 4.61 (dd, *J* = 8.7, 5.9 Hz, 1H), 3.54 – 3.40 (m, 2H), 2.74 (s, 3H), 2.49 (d, *J* = 0.8 Hz, 3H), 1.74 (d, *J* = 0.9 Hz, 3H), 1.53 (s, 9H). MS (ESI) for C_26_H_29_N_5_O_3_S [M+H]^+^: m/z calcd, 492.61; found 492.30.

**tert-butyl (S)-2-(4-(4-aminophenyl)-2,3,9-trimethyl-6H-thieno[3,2-f][1,2,4]triazolo[4,3-a][1,4]diazepin-6-yl)acetate (MMH248)**

**MMH248** was prepared according to the procedure previously described by Dragovich *et al*. with slight modifications.^1^ Briefly, a mixture of JQ1 (1.40 g, 3.06 mmol), diphenylmethanimine (1.055 g, 5.82 mmol), Xphos-PdG2 (0.241 g, 0.306 mmol), and cesium carbonate (2.994 g, 9.19 mmol) in toluene (20 mL) was heated at 110 ^o^C and left to stir overnight. The next day, the mixture was cooled to room temperature, filtered, and the filtrate was concentrated under vacuum. The residue was purified by flash column chromatography (EtOAc:hexane) and the benzophenone-imine protected product identity was confirmed by LCMS. MS (ESI) for C_36_H_36_N_5_O_2_S [M+H]^+^: m/z calcd, 602.77; found 602.40. The imine was hydrolyzed by the addition of 20 mL THF, and adding 5 mL of 1 M HCl, followed by stirring for approximately 10 minutes at room temperature. The mixture was dried under vacuum to yield **MMH248** (927 mg, 2.12 mmol, overall yield 69.2 %). ^1^H NMR (500 MHz, DMSO) δ 7.12 (d, *J* = 8.1 Hz, 2H), 6.52 (d, *J* = 8.9 Hz, 2H), 2H), 5.62 (s, 2H), 4.30 (dd, *J* = 8.3, 6.3 Hz, 1H), 3.30 – 3.21 (m, 2H), 2.58 (s, 3H), 2.43 (d, *J* = 1.0 Hz, 3H), 1.74 (d, *J* = 0.9 Hz, 2H), 1.42 (s, 9H). MS (ESI) for C_23_H_27_N_5_O_2_S [M+H]^+^: m/z calcd, 438.57; found 438.34. MS (ESI) for C_23_H_28_N_5_O_2_S [M+H]^+^: m/z calcd, 438.56; found 438.35.

**tert-butyl (S)-2-(4-(4-acrylamidophenyl)-2,3,9-trimethyl-6H-thieno[3,2-f][1,2,4]triazolo[4,3-a][1,4]diazepin-6-yl)acetate (MMH1)**

**MMH1** was prepared by adding acryloyl chloride (4.5 µL, 0.055 mmol) to a mixture of **MMH248** (20.0 mg, 0.046 mmol) and DIPEA (23.4 µL, 0.137 mmol) stirred in 2 mL dichloromethane. The reaction was stirred for 5 minutes and monitored by LCMS, and subsequently quenched with methanol followed by drying under vacuum. The mixture was reconstituted in methanol and purified by prep HPLC (method 2) to give **MMH1** (1.40 mg, 0.00285 mmol, 6.23% yield). ^1^H NMR (500 MHz, DMSO) δ 10.35 (s, 1H), 7.71 (d, *J* = 9.0 Hz, 2H), 7.40 (d, *J* = 8.4 Hz, 2H), 6.44 (dd, *J* = 16.9, 10.1 Hz, 1H), 6.28 (dd, *J* = 16.9, 2.0 Hz, 1H), 5.78 (dd, *J* = 10.1, 2.0 Hz, 1H), 4.39 (dd, *J* = 8.2, 6.3 Hz, 1H), 3.35 – 3.25 (m, 2H), 2.61 (s, 3H), , 2.43 (s, 3H), 1.67 (s, 3H), 1.43 (s, 9H). MS (ESI) for C_26_H_30_N_5_O_3_S [M+H]^+^: m/z calcd, 492.61; found 492.35.

**tert-butyl (S)-2-(2,3,9-trimethyl-4-(4-propionamidophenyl)-6H-thieno[3,2-f][1,2,4]triazolo[4,3-a][1,4]diazepin-6-yl)acetate (MMH1-NR)**

Propionyl chloride (2.5 µL, 0.0282 mmol) was added to a mixture containing **MMH248** (10.3 mg, 0.0235 mmol) and DIPEA (8.05 µL, 0.0471 mmol) in 2 mL dichloromethane. The reaction was left to stir for 1 h, before evaporation under vacuum. The mixture was redissolved in methanol and purified by prep HPLC (method 2) to give **MMH1-NR** (7.75mg, 0.0157 mmol, 66.8% yield). ^1^H NMR (500 MHz, DMSO) δ 10.08 (s, 1H), 7.64 (d, *J* = 9.1 Hz, 2H), 7.36 (d, *J* = 8.4 Hz, 2H), 4.39 (dd, *J* = 8.2, 6.3 Hz, 1H), 3.38 – 3.24 (m, 2H), 2.61 (s, 3H), 2.43 (d, *J* = 0.9 Hz, 3H), 2.34 (q, *J* = 7.6 Hz, 2H), 1.66 (d, *J* = 1.0 Hz, 3H), 1.43 (s, 9H), 1.08 (t, *J* = 7.6 Hz, 3H). MS (ESI) for C_26_H_32_N_5_O_3_S [M+H]^+^: m/z calcd, 494.63; found 494.42.

**tert-butyl (S)-2-(2,3,9-trimethyl-4-(4-(vinylsulfonamido)phenyl)-6H-thieno[3,2-f][1,2,4]triazolo[4,3-a][1,4]diazepin-6-yl)acetate (MMH2)**

Ethenesulfonyl chloride (23.9 µL, 0.263 mmol) was added to a mixture of **MMH248** (9.59 mg, 0.0219 mmol) and pyridine (35.5 µL, 0.438 mmol). The reaction was instantaneous as shown by LCMS. The reaction was washed once with 1 M HCl (1 mL), then the remaining DCM mixture was evaporated. The mixture was reconstituted in methanol to quench excess electrophile, and was subsequently purified by prep HPLC (method 2) to give **MMH2** (5.94 mg, 0.0113 mmol, 51.4% yield). ^1^H NMR (500 MHz, DMSO-*d*_6_) δ 10.32 (s, 1H), 7.36 (d, *J* = 8.4 Hz, 2H), 7.17 (d, *J* = 8.85 2H), 6.79 (dd, *J* = 16.4, 10.0 Hz, 1H), 6.14 (d, *J* = 16.4 Hz, 1H), 6.05 (d, *J* = 9.9 Hz, 1H), 4.39 (dd, *J* = 8.3, 6.3 Hz, 1H), 3.38 – 3.24 (m, 2H), 2.60 (s, 3H), 2.42 (s, 3H), 1.65 (s, 1H), 1.43 (s, 9H). MS (ESI) for C_25_H_30_N_5_O_4_S_2_ [M+H]^+^: m/z calcd, 528.67; found 528.21.

**tert-butyl (S)-2-(4-(4-(ethylsulfonamido)phenyl)-2,3,9-trimethyl-6H-thieno[3,2-f][1,2,4]triazolo[4,3-a][1,4]diazepin-6-yl)acetate (MMH2-NR)**

To a solution of 4 mL of ethanol, was added **MMH2** (2.26 mg, 0.00428 mmol) and 10% palladium on carbon (10.5 mg, 0.00985 mmol), followed by purging with hydrogen gas via a balloon for 10 min. The reaction was subsequently stirred under hydrogen for another 10 min, flushed through celite, then evaporated and lyophilized to quantitatively yield **MMH2-NR** (2.26 mg, 0.00427 mmol, 99.7% yield). ^1^H NMR (500 MHz, DMSO) δ 10.09 (s, 1H), 7.37 (d, *J* = 8.5 Hz, 2H), 7.23 (d, *J* = 9.0 Hz, 2H), 4.39 (dd, *J* = 8.3, 6.2 Hz, 1H), 3.33 – 3.24 (m, 2H), 3.12 (q, *J* = 7.3 Hz, 2H), 2.59 (s, 3H), 2.42 (s, 3H), 1.67 (s, 3H), 1.43 (s, 9H), 1.16 (t, *J* = 7.3 Hz, 3H). MS (ESI) for C_25_H_32_N_5_O_4_S_2_ [M+H]^+^: m/z calcd, 530.68; found 530.33.

**tert-butyl (S)-2-(4-(4-(2-chloroacetamido)phenyl)-2,3,9-trimethyl-6H-thieno[3,2-f][1,2,4]triazolo[4,3-a][1,4]diazepin-6-yl)acetate (MMH249)**

To a solution of **MMH248** (40 mg, 0.0914 mmol), chloroacetic acid (21.6 mg, 0.229 mmol), and DIPEA (47 µL, 0.274 mmol) in 3 mL DMF, was added HATU (104 mg, 0.274 mmol). The reaction was purified by HPLC (method 2) to yield **MMH249** which was again purified by column chromatography using DCM and methanol (14 mg, 29.8 % yield). ^1^H NMR (500 MHz, DMSO) δ 10.51 (s, 1H), 7.64 (d, *J* = 8.7 Hz, 2H), 7.40 (d, *J* = 8.5 Hz, 2H), 4.40 (dd, *J* = 8.2, 6.3 Hz, 1H), 4.27 (s, 2H), 3.33 – 3.27 (m, 2H), 2.60 (s, 3H), 2.43 (d, *J* = 0.9 Hz, 3H), 1.66 (d, *J* = 0.9 Hz, 3H), 1.43 (s, 9H). MS (ESI) for C_25_H_28_ClN_5_O_3_S [M+H]^+^: m/z calcd, 514.16 and 516.16 for ^35^Cl and ^37^Cl respectively; found 514.26 and 516.21 respectively.

**Spectral Characterization of Final Compounds (^1‑^H NMR, LC, and MS Spectra)**

**(*S*)-GNE011 (500 MHz ^1^H NMR in DMSO-*d*_6_)**

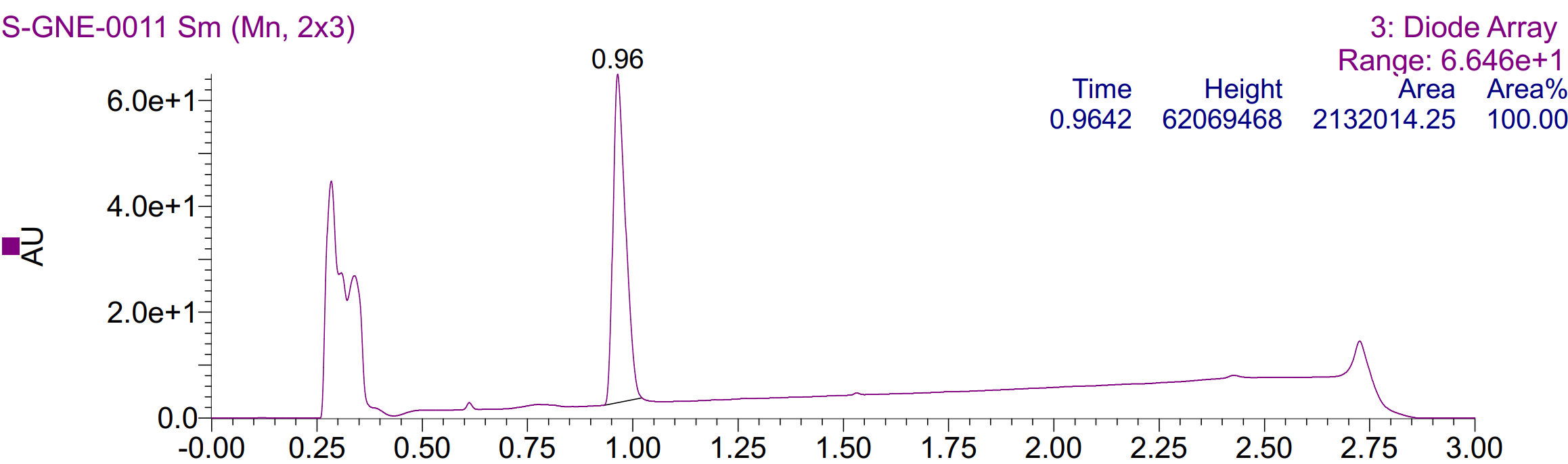


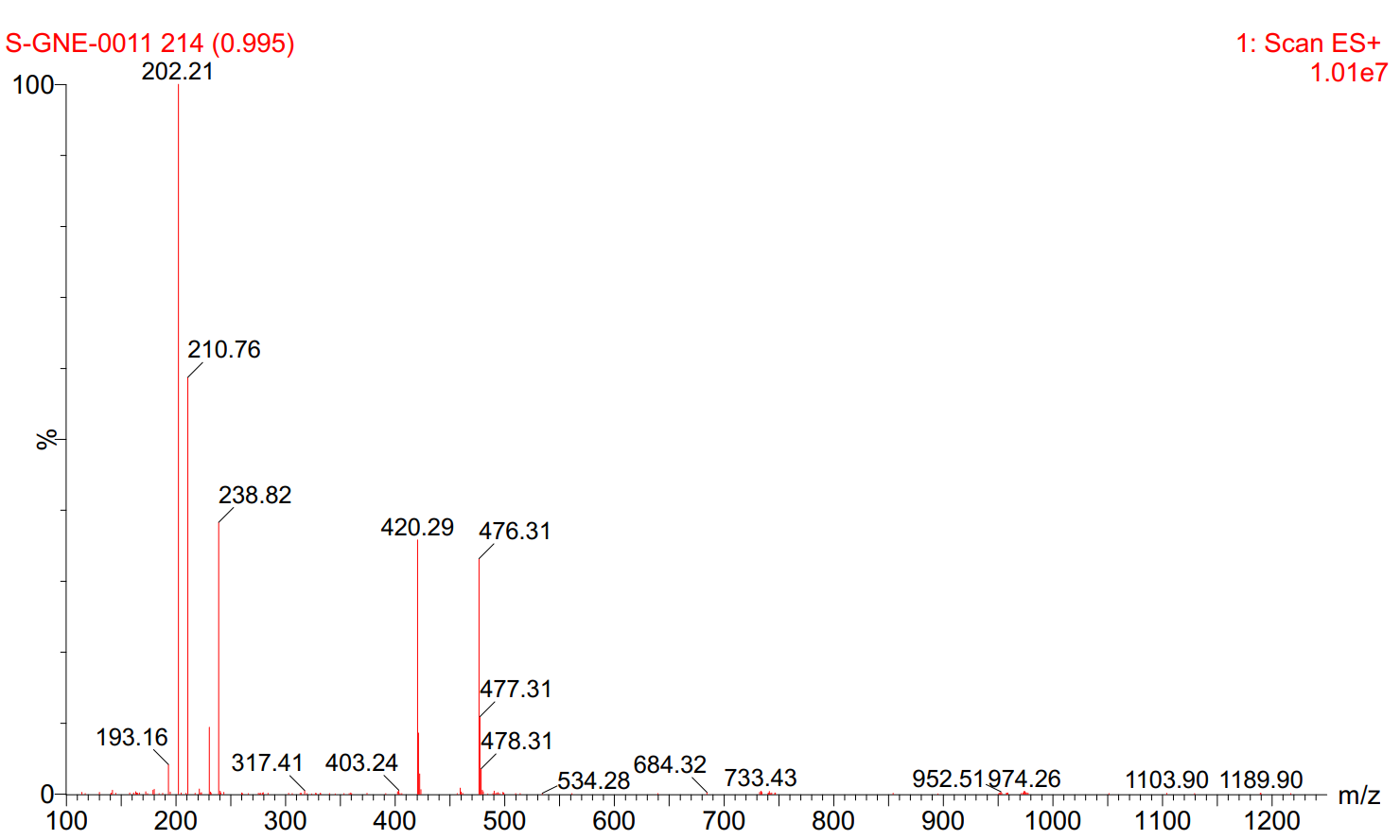


**TMX1 (500 MHz ^1^H NMR in DMSO-*d*_6_)**

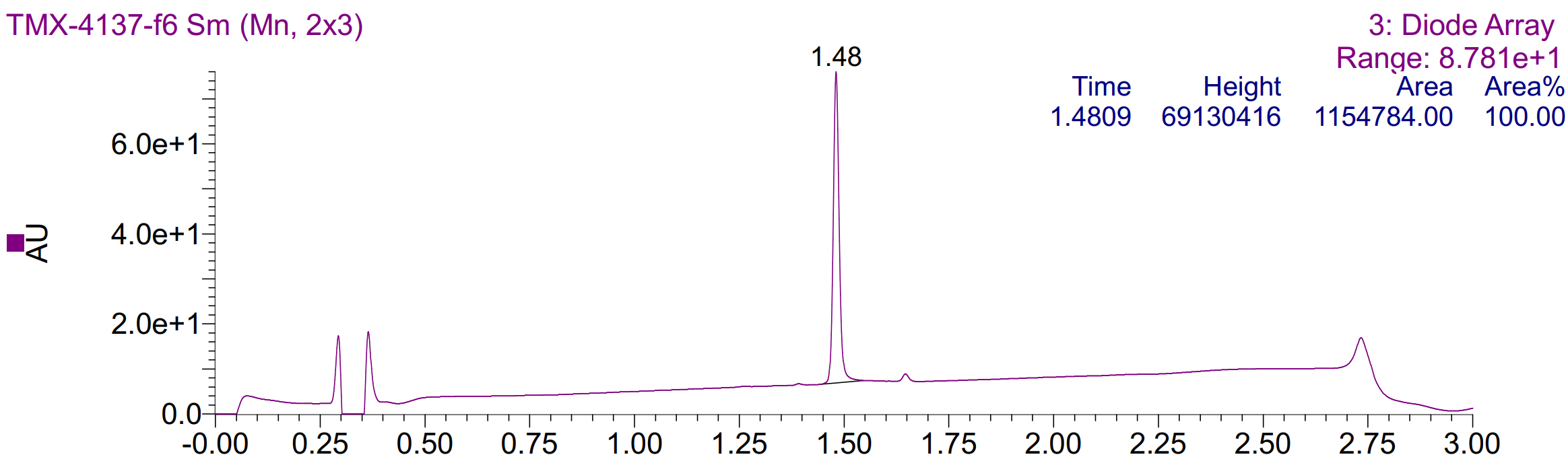


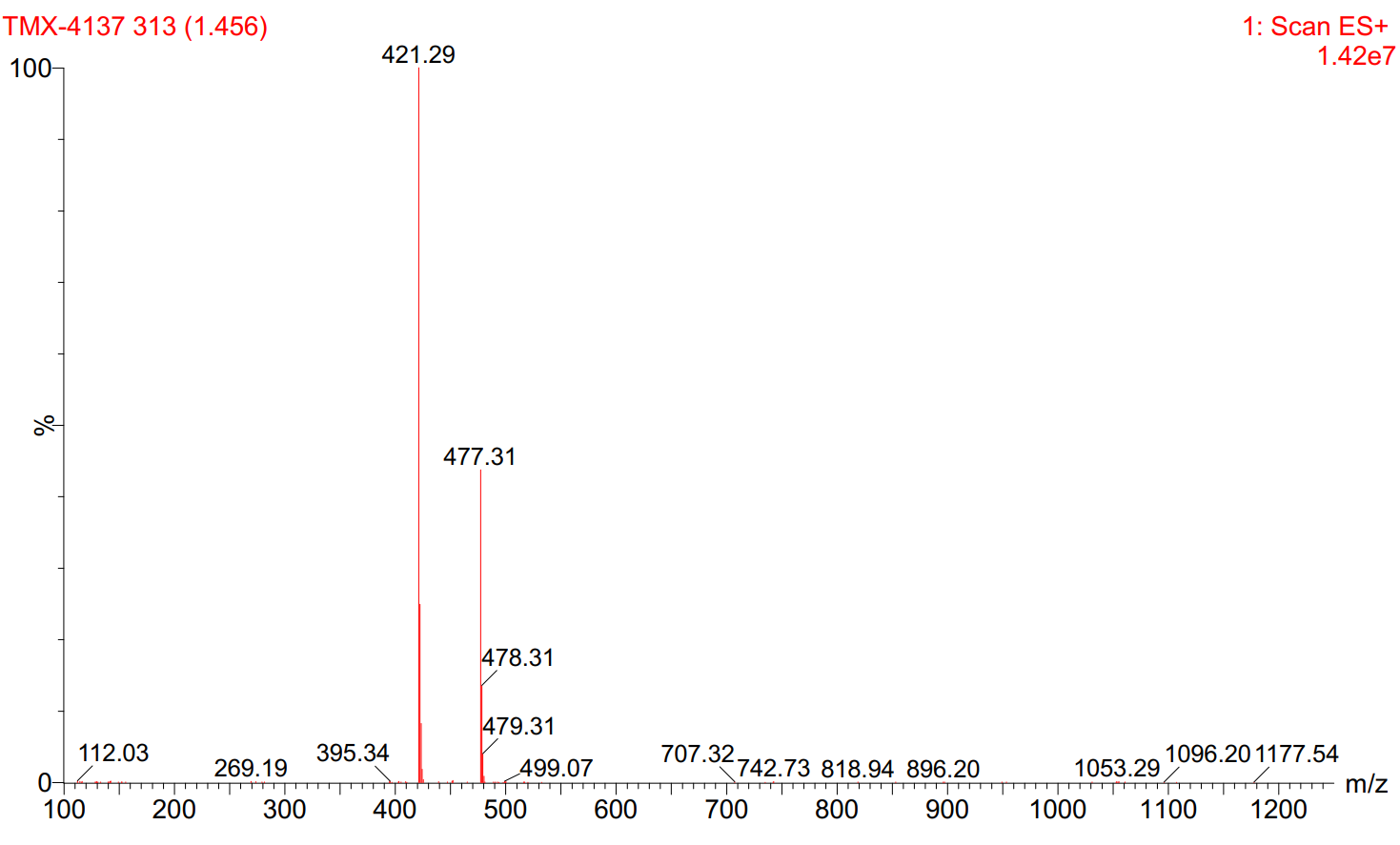


**MMH1 (500 MHz ^1^H NMR in DMSO-*d*_6_)**

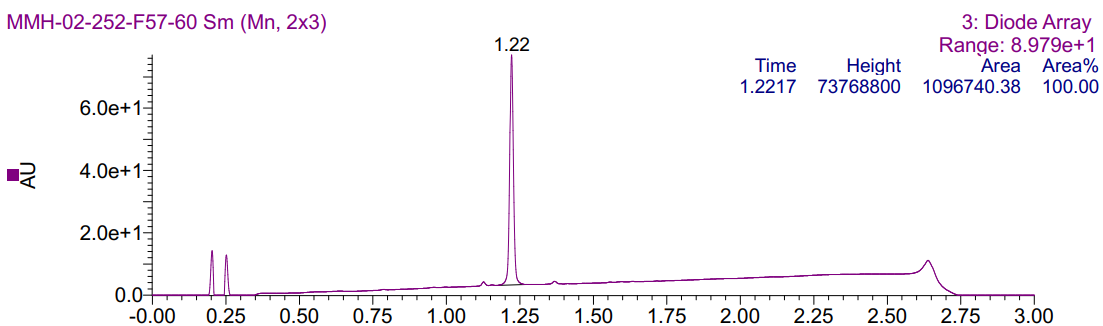


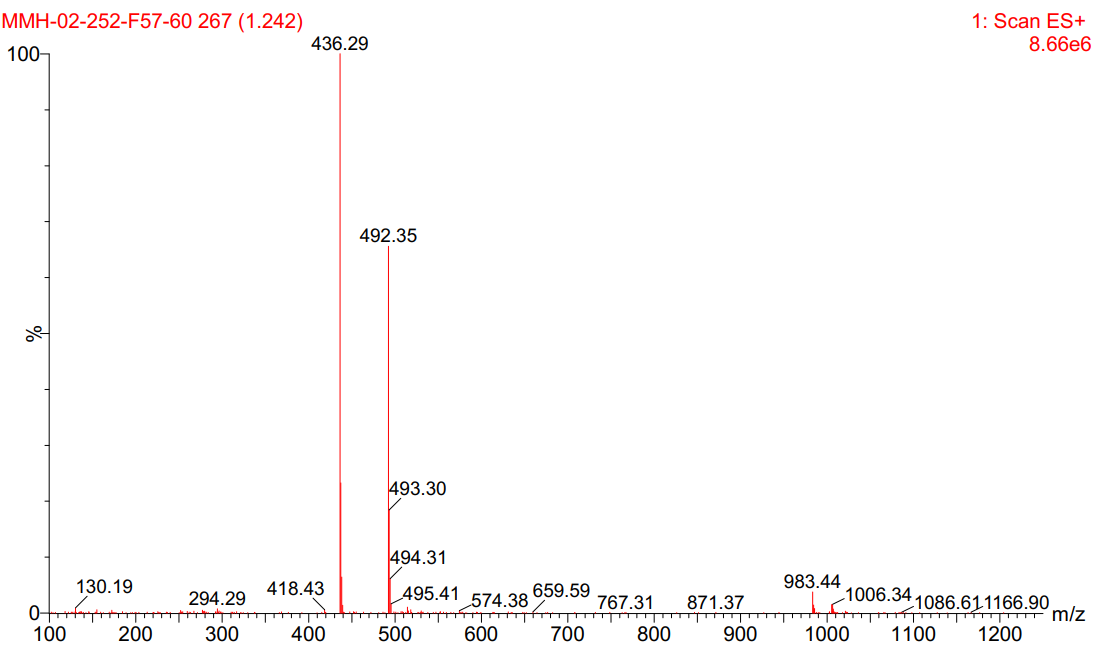


**MMH1-NR (500 MHz ^1^H NMR in DMSO-*d*_6_)**

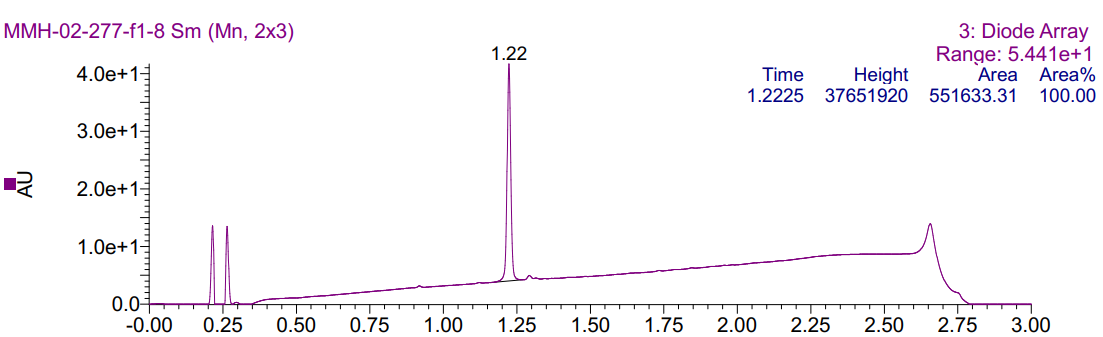


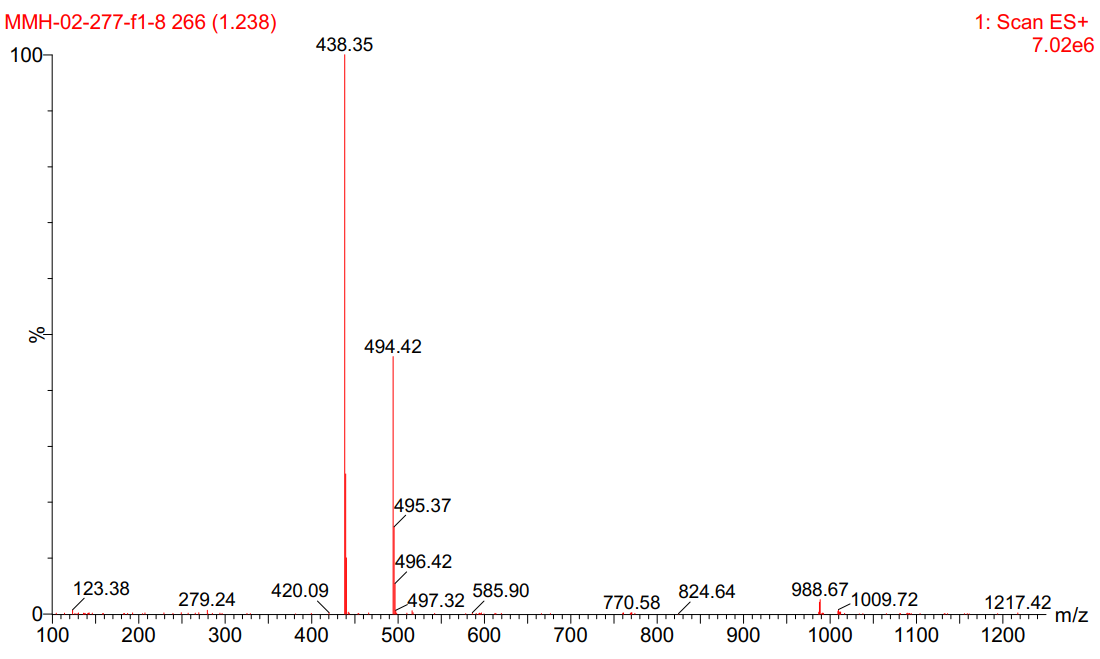


**MMH2 (500 MHz ^1^H NMR in DMSO-*d*_6_)**

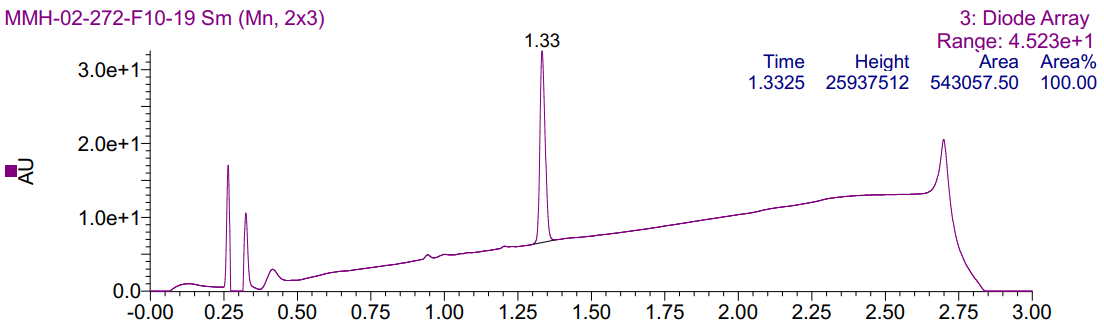


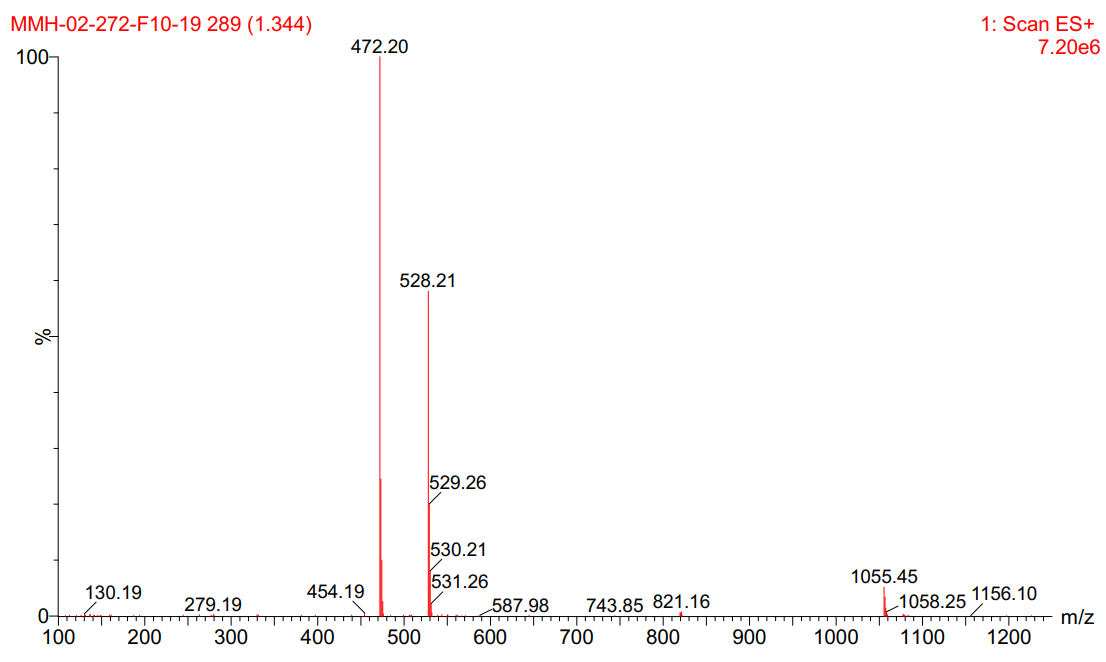


**MMH2-NR (500 MHz ^1^H NMR in DMSO-*d*_6_)**

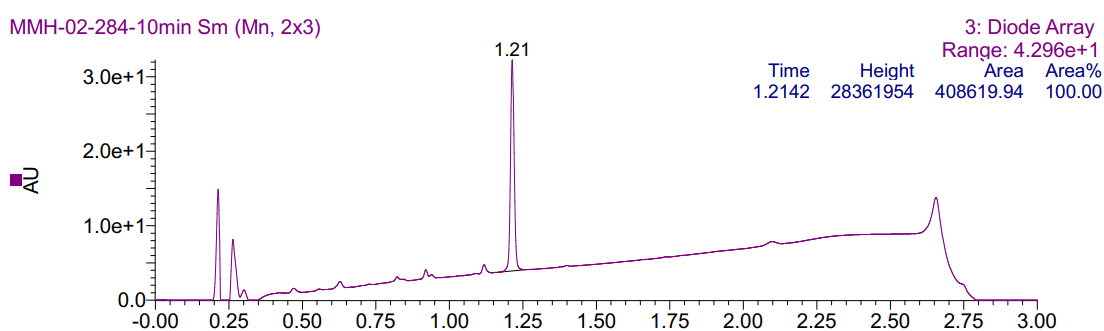


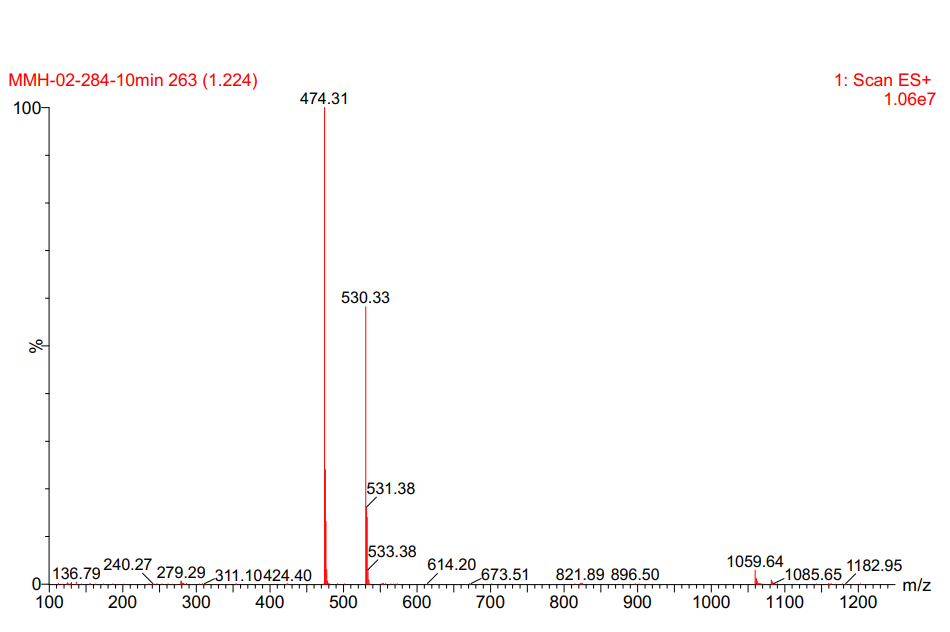


**MMH248 (500 MHz ^1^H NMR in DMSO-*d*_6_)**

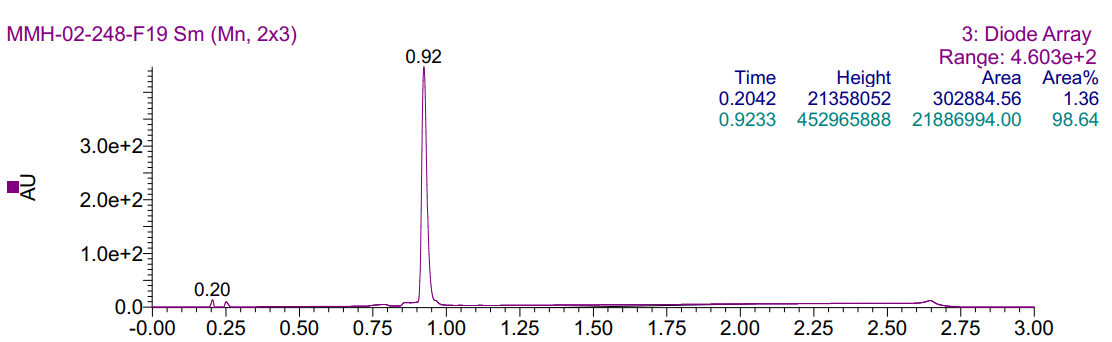


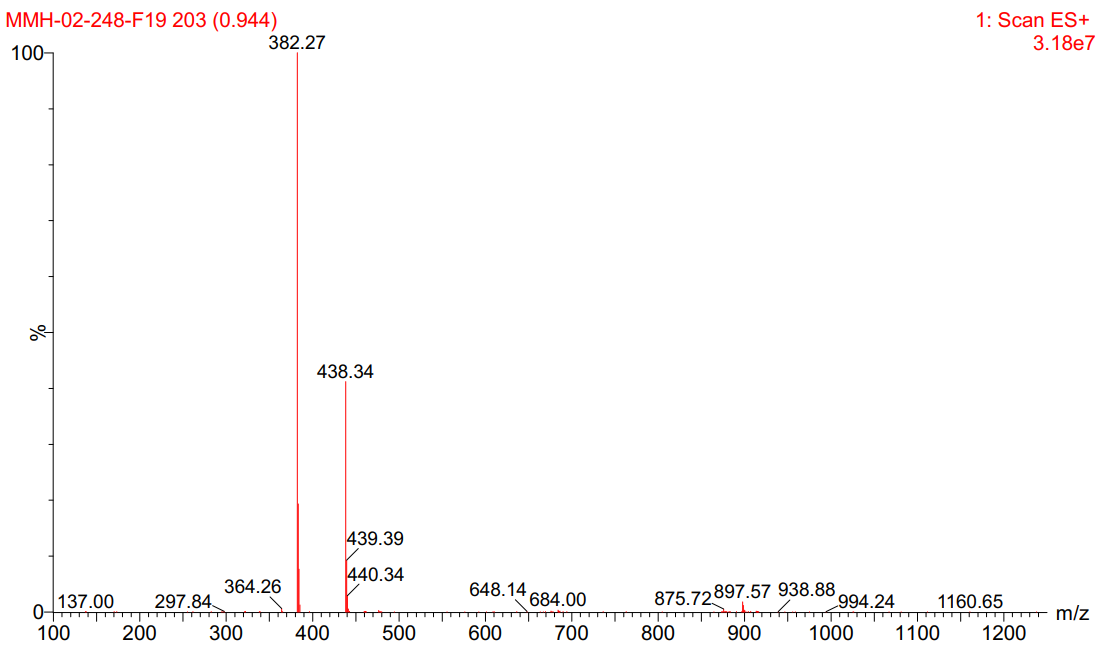


**MMH249 (500 MHz ^1^H NMR in DMSO-*d*_6_)**

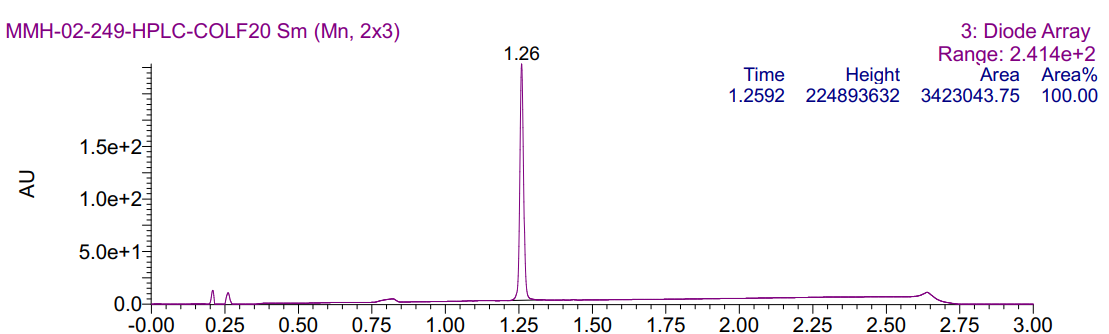


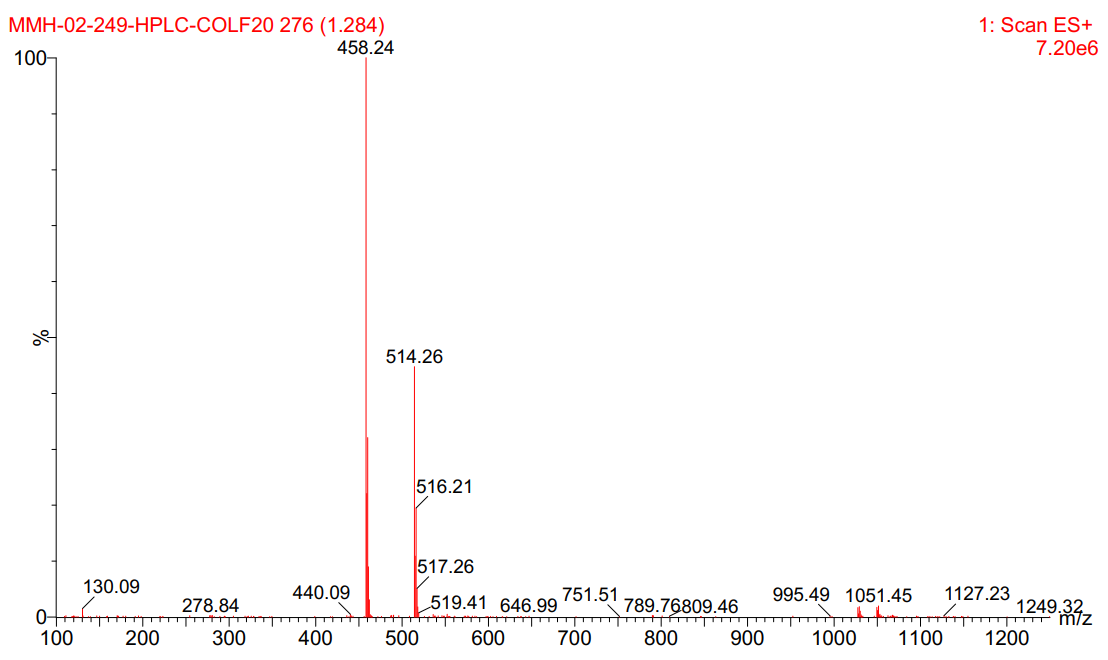


**MMH263 (500 MHz ^1^H NMR in DMSO-*d*_6_)**

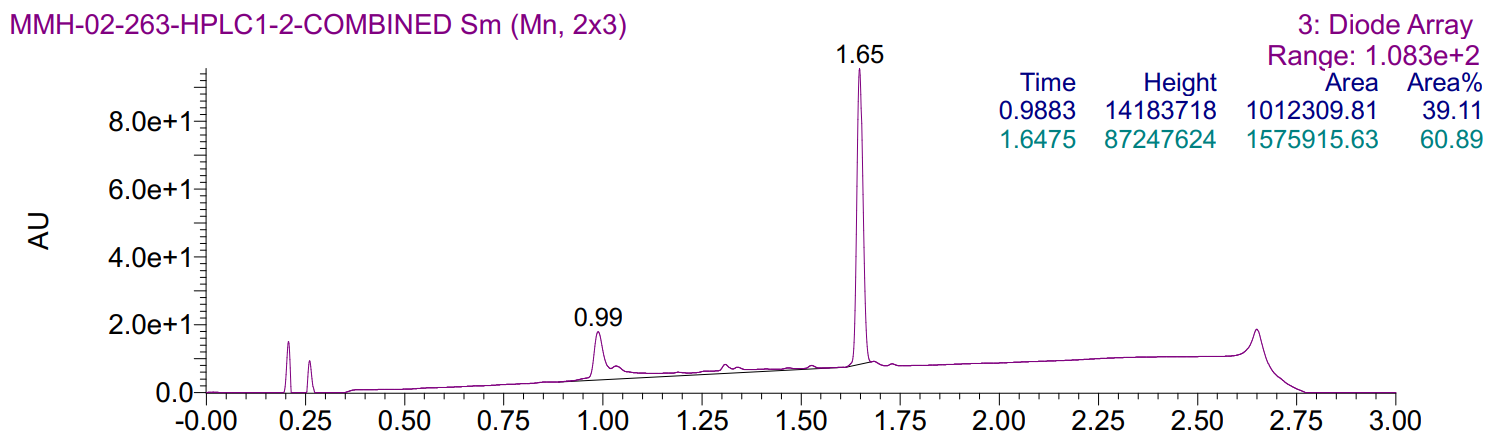


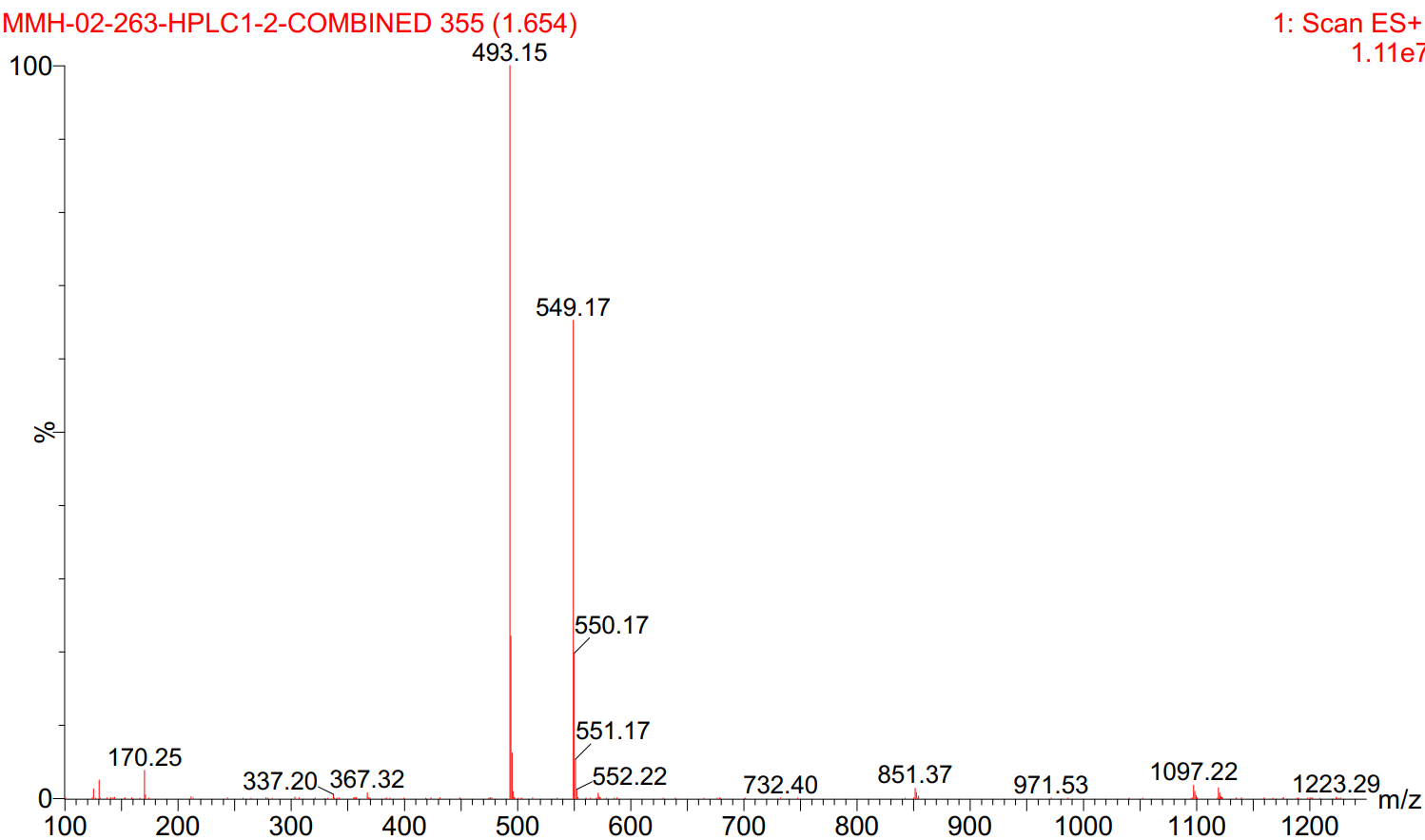


**MMH269 (500 MHz ^1^H NMR in DMSO-*d*_6_)**

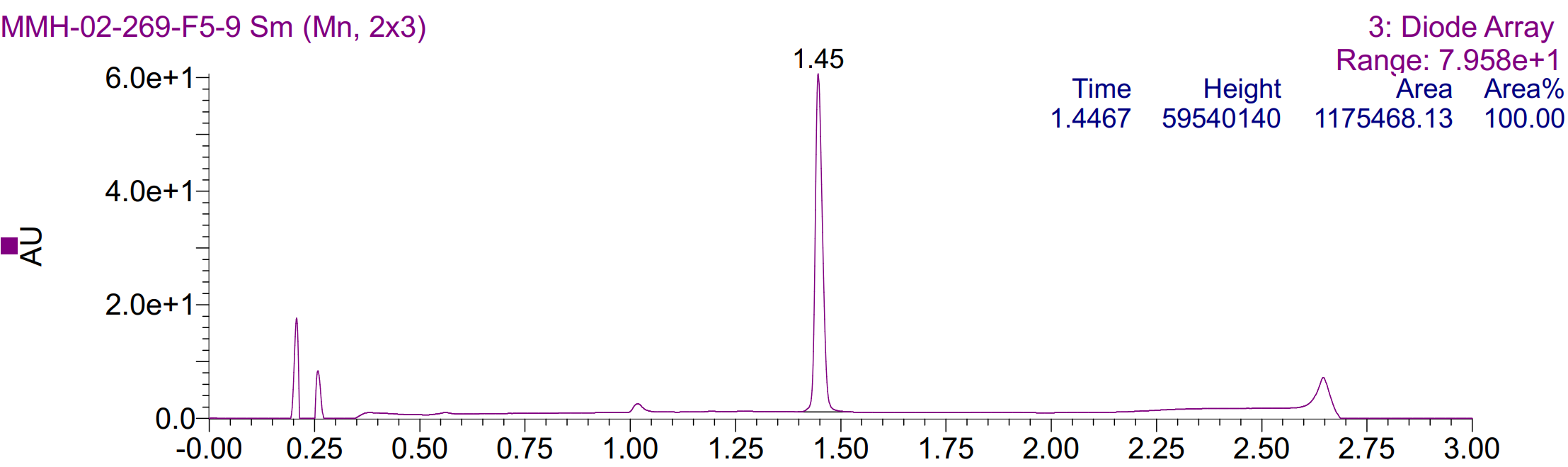


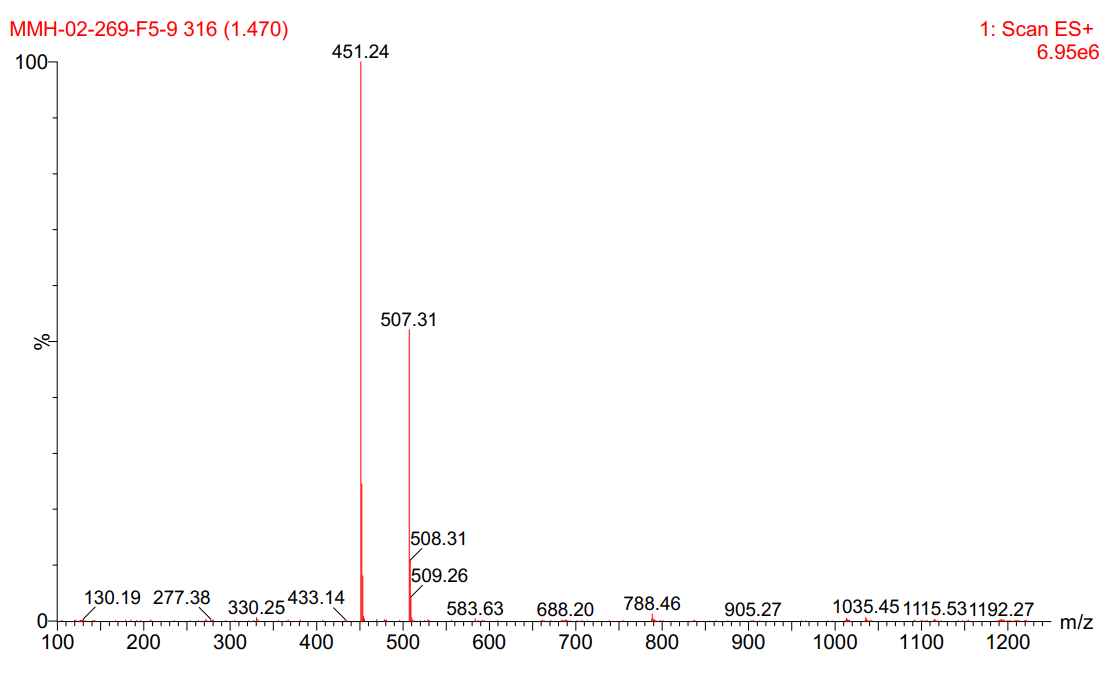


**MMH271 (500 MHz ^1^H NMR in DMSO-*d*_6_)**

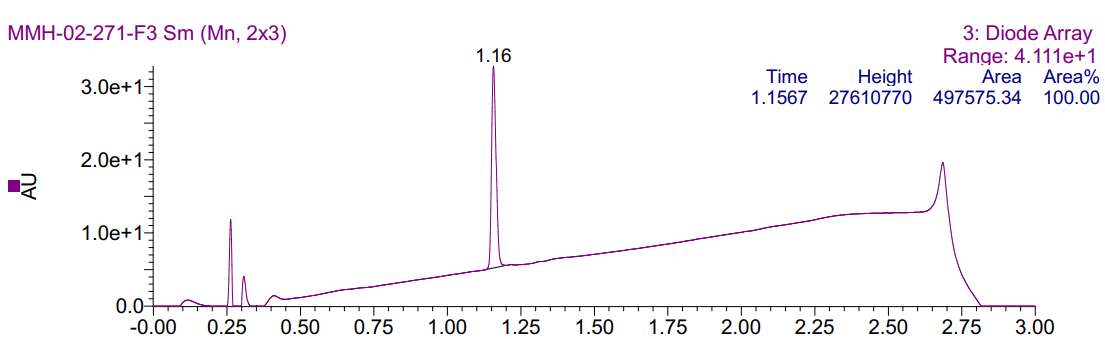


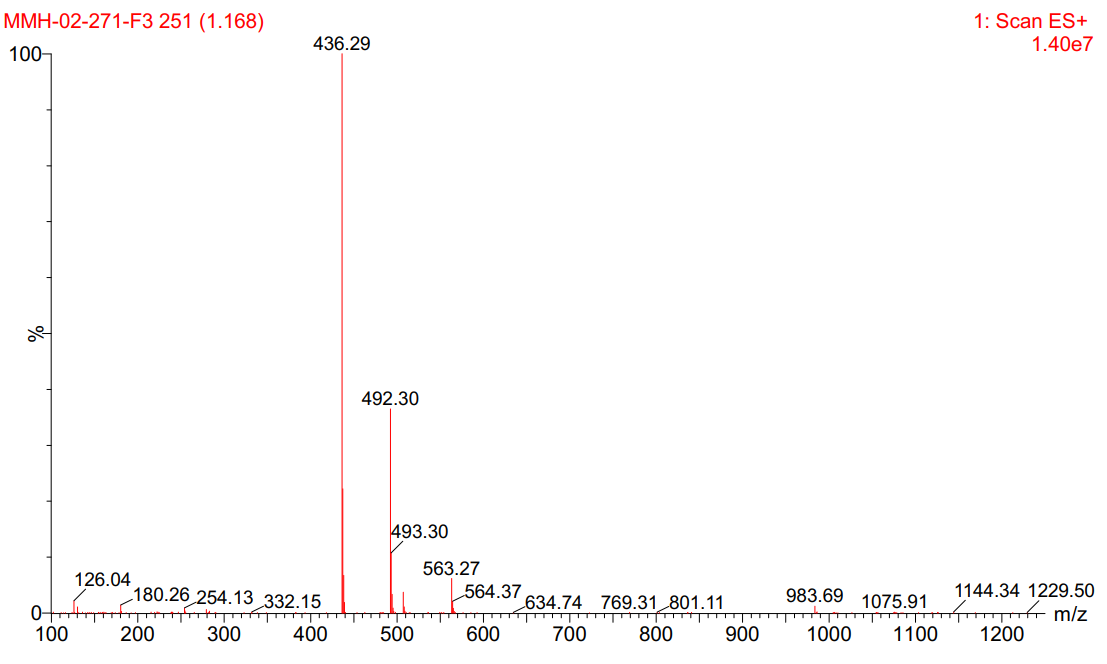


**MMH287 (500 MHz ^1^H NMR in DMSO-*d*_6_)**

**
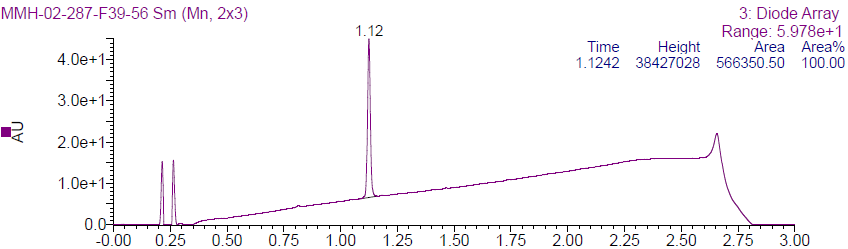
** **
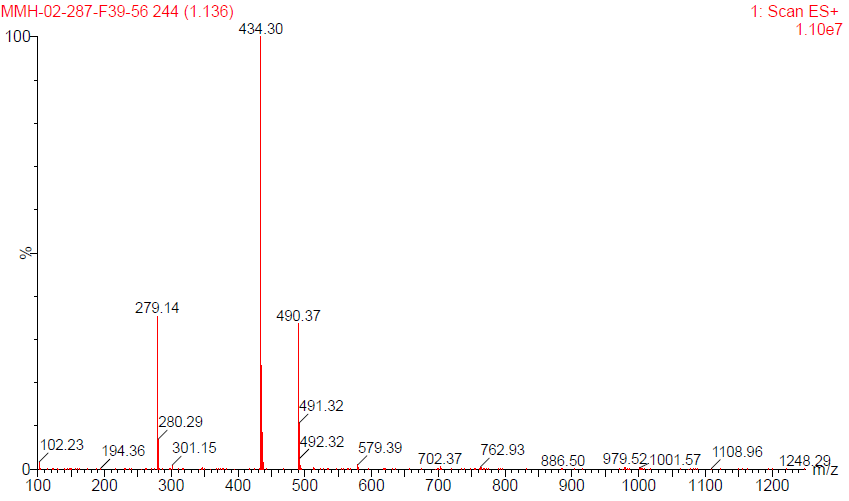
**

**TMX458 (500 MHz ^1^H NMR in DMSO-*d*_6_)**

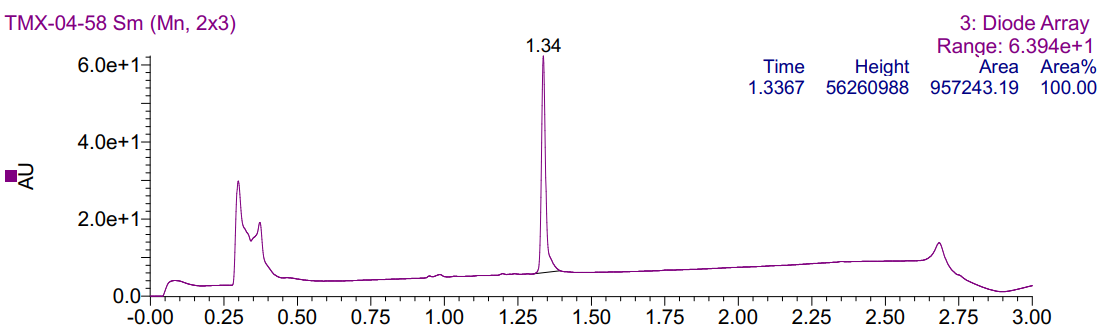


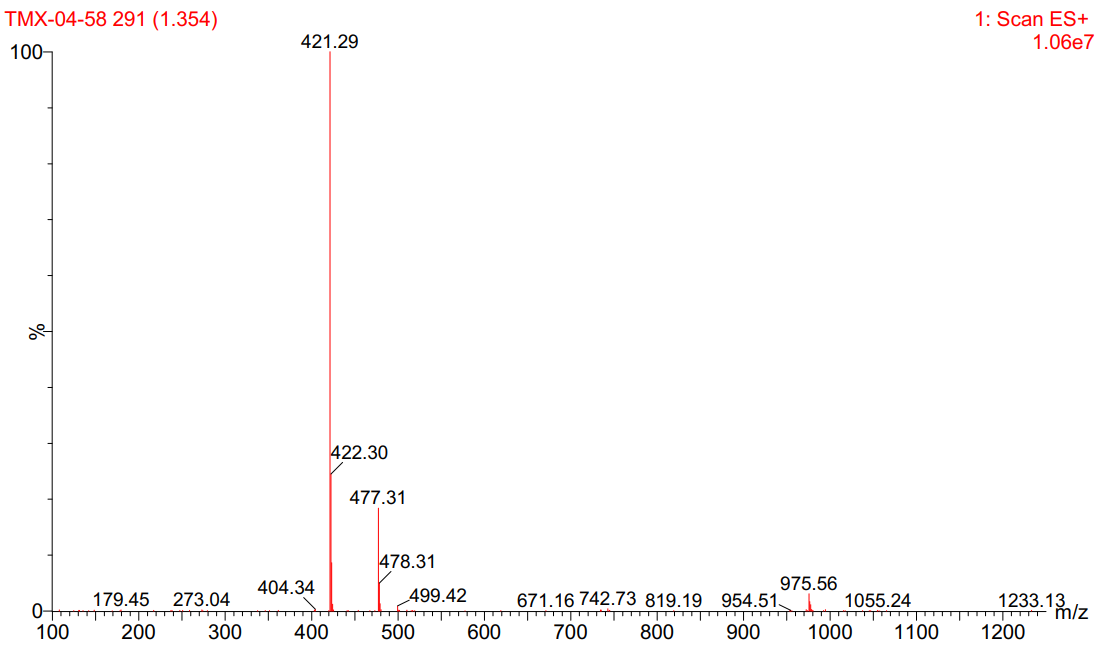


**TMX4128 (500 MHz ^1^H NMR in DMSO-*d*_6_)**

**
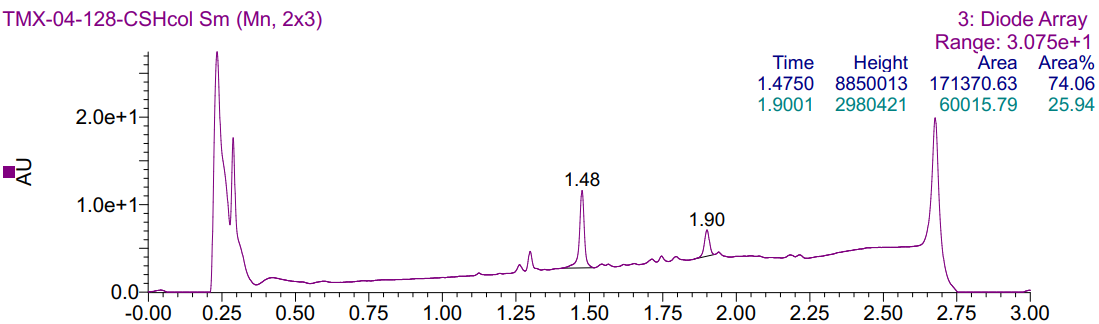
**

**
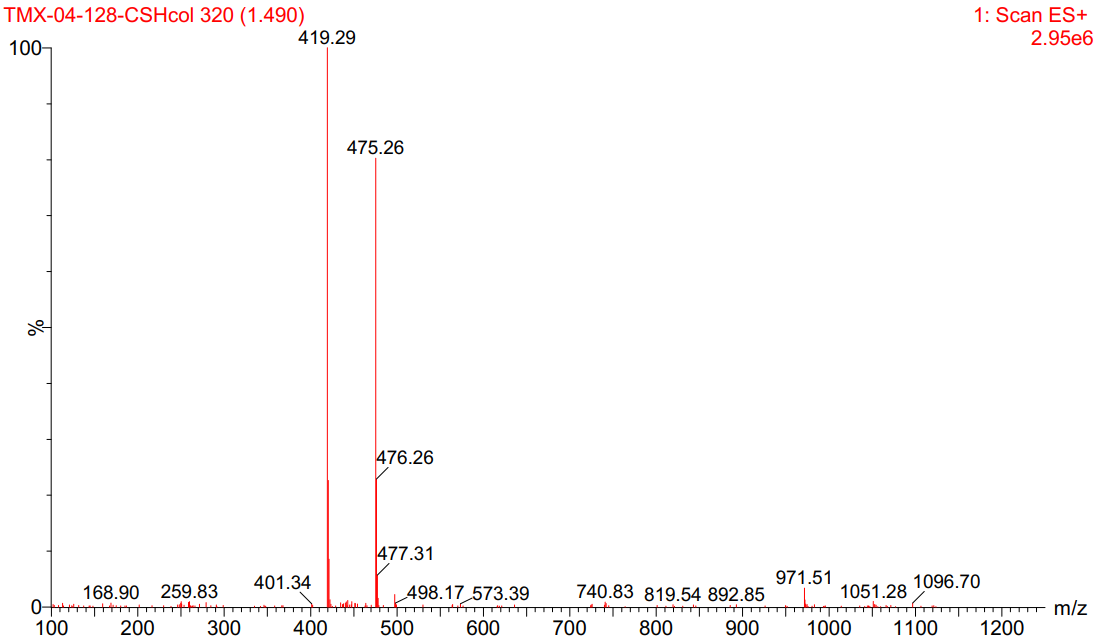
**

**References**

1. Dragovich, P. S. *et al.* Antibody-Mediated Delivery of Chimeric BRD4 Degraders. Part 1: Exploration of Antibody Linker, Payload Loading, and Payload Molecular Properties. *J. Med. Chem.* **64**, 2534–2575 (2021).
